# Mitigating the harmful consequences of arsenic on celosia plants via the use of GABA

**DOI:** 10.1186/s12870-026-08384-2

**Published:** 2026-03-13

**Authors:** Amr S. Mohamed, Asmaa E. Abd Elhafez, Mayada M. N. Ahmad

**Affiliations:** 1 Botanical Gardens Research Dept., Horticulture Research Institute, Agricultural Research Center, Giza/Alexandria, Egypt; 2 Ornamental Plants and Woody Trees Department, National Research Centre, Dokki, Giza, Egypt

**Keywords:** Arsenic, *Celosia argentea var. Spicata* L.,, GABA, Metalloid, ROS

## Abstract

**Background:**

Arsenic (As) is a highly toxic metalloid that severely disrupts plant growth, metabolic function, and productivity by inducing oxidative stress and disturbing nutrient homeostasis. Soil contamination with arsenic severely limits plant growth and reduces the ornamental value of many species. In the present study, *Celosia argentea var. spicata* was grown in soil containing different arsenic levels (0, 30, 50, and 70 mg kg⁻¹ soil) to examine the ability of γ-aminobutyric acid (GABA) to mitigate arsenic-induced stress in both seasons (2023 and 2024).

**Results:**

Our results revealed that exposure to As markedly reduced vegetative and reproductive growth characteristics—such as plant height, branch number, leaf area, and both fresh and dry biomass—and that these inhibitory effects intensified with increasing As levels. In contrast, exogenous GABA individually had significantly improved all growth and flowering parameters, at interaction conditions 1 mM treatment producing the greatest increase even under severe arsenic stress. At the physiological level, GABA application increased photosynthetic pigment concentrations (chlorophyll a, chlorophyll b, and carotenoids), total soluble sugars, and flavonoids while decreasing the accumulation of proline and phenolic compounds. GABA also reduced the As content in shoots and soil while promoting its retention within root tissues, suggesting a protective mechanism of immobilization. Biochemically, GABA alleviated oxidative damage by substantially lowering malondialdehyde (MDA) and hydrogen peroxide (H₂O₂) levels, with the strongest effects observed at 1 mM GABA. Exogenous γ-aminobutyric acid (GABA) application significantly enhanced superoxide dismutase (SOD) isoenzyme activity under arsenic-induced stress conditions.

**Conclusions:**

The results demonstrate that exogenous GABA at 0.5 or 1 mM can play a meaningful role in restricting arsenic movement from roots to shoots inside plants and reducing the oxidative damage associated with arsenic stress in *C. argentea* var. *spicata*.

**Supplementary Information:**

The online version contains supplementary material available at 10.1186/s12870-026-08384-2.

## Background

 The genus *Celosia* contains ornamental as well as edible herbaceous annuals and perennials. It belongs to the amaranth family (*Amaranthaceae*). It is native to Africa, North America and South America. *Celosia* has three varieties whose bloom shapes differ. The inflorescences of *Celosia argentea var. cristata* are cockscomb- and crested-type blooms that look brain- or coral-shaped. However, *C. argentea var. plumosa* generates flame- or plume-shaped blooms. Finally, *C. argentea var. spicata* produces spiked-, feather-, or wheat-shaped inflorescences. The most suitable varieties of *Cristata* cultivars are containers and pots, whereas the use of Spicata and Plumosa varieties is more advisable for fresh flower arrangements [1]. *Celosia spicata* (*C. argentea* var. *spicata*) is an ornamental summer annual that is distinguished by its discriminatory, erect spikes. These spikes resemble the sheaves of wheat; therefore, the Spicata common name is wheat celosia. Abundant erect cylindrical inflorescences emerge on the branch tips above the foliage from mid-summer. The colors of the tip florets are red, pink, purple or bored; then, over time, these colors mostly change to a metallic silver sheen on the calyxes and remain after the flowers wither. Wheat celosia could be in annual or mixed beds and borders for either vertical interest or as a contrast with mounded forms [2]. *C. argentea* is nominated as a heavy metal hyperaccumulator plant, such as cadmium and zinc. It also phyto-remediates heavily contaminated soils containing these elements by storing them up in its various parts [3, 4]. However, there is no available information about how this plant reacts to soils contaminated by arsenic.

Arsenic (As) is a metalloid found in soil in two prevalent inorganic forms: arsenate and arsenite. It is considered a toxic environmental pollutant, whereas it is a nonessential element because it has no vital function in the body; in contrast, it causes serious health risks, so it is grouped as a major pollutant by the Global Environmental Protection Agency. Thence, the Agency for Toxic Substances and Disease Registry and the United States Environmental Protection Agency (US EPA) count As among the top 20 severe substances [5]. The concentrations of As in the environment rise as a result of either natural sources (rock weathering, volcanic emissions and discharge from hot springs) or anthropogenic activities (the use of arsenicals as pesticides and herbicides, mining processes, smelting and wood preservatives). Moreover, it is highly mobile and bioavailable to plants. As negatively influences several main cellular processes, such as ATP synthesis and oxidative phosphorylation. In addition, it interrupts nutrient uptake and distribution by vieing with nutrients, causing disorders in the metabolic processes of plants [6, 7].

Gamma-aminobutyric acid (GABA) is a nonprotein amino acid formed from four water-soluble carbons. It is a signaling molecule that interferes with many physiological and metabolic regulations. Its signaling role in plants is adjusted by its interplay with aluminum-activated malate transporters (ALMTs), which modify anion flux and root growth under stress. Moreover, GABA facilitates increased redox and osmotic homeostasis, cytosolic pH regulation, root growth, reproductive development, and cell wall remodeling, and it intercedes with plastid modifications. These responses organize the mechanisms that are associated with growth-to-senescence and have a remarkable ability to alleviate environmental stressors in plants by inducing acclimatization mechanisms. Furthermore, GABA has antioxidant properties that efficiently counteract reactive oxygen species (ROS), and thus, it protects cells from oxidative fatigue under stress conditions. All these abilities provide plants with a chance to survive under stress conditions [8, 9]. It has been reported that the GABA precursor 5-aminolevulinic acid (ALA), which arises from glutamate, has a valuable influence on reducing As toxicity in terms of photosynthetic activity and oxidative damage; on the other hand, it stimulates the defense system under As stress in wheat plants. GABA influences phosphorus retention and carbon metabolism in plants grown under As stress, thereby sustaining growth and mitigating stress effects [9].

Although we have a good understanding of GABA’s various key roles in mitigating different abiotic stresses, its ability to alleviate the toxicity of As is still poorly understood. Moreover, the information about its effects on As accumulation and translocation in ornamental plants like *C. spicata* is rare. This gap also includes the impacts on their ornamental traits, such as flowering and pigmentation. Therefore, we hypothesized that exogenous GABA would mitigate As toxicity in Celosia by enhancing antioxidant defense and regulating As sequestration in roots.

Thus, this experiment was performed to test the ability of GABA to enhance the growth and flowering of *Celosia argentea* var. *spicata* plants grown in soils polluted with different concentrations of arsenic.

## Materials and methods

### Location and duration

The trial was conducted during the period from March to August, within two subsequent seasons (2023 and 2024) at the nursery of the Experimental Research Station of National Research Centre in the Al-Nubaria region, Al Bahira Governorate, Egypt, 80 km east of Cairo, and its location is latitude 30° 30’ 1.4‘N and longitude 30° 19’ 10.9’ E.

### Plant material

*Celosia argentea. var. spicata* L. seeds were secured from Horticulture Research Institute, Agricultural Research Center, Giza, Egypt.

### Procedure and layout of the experiment

The seeds were cultured in plastic trays on the 2nd of March 2023 and 2024. Germination started after the seeds were cultured for ten days. Seedlings (10 cm in length and carrying 6 pairs of leaves) were individually transplanted on the 18th of May in both seasons in a plastic pot (30 cm in diameter) filled with 6 kg of growing media consisting of silt and sand at a ratio of 1:1 (v/v). The layout of the experiment was a complete randomized block design. The trial included 12 treatments, and every treatment included 10 replicates, i.e., 10 pots for each treatment with one plant for each pot.

The 12 treatments were as follows: (1) As 0 mg/kg + 0 mM GABA; (2) As 0 mg/kg + 0.5 mM GABA; (3) As 0 mg/kg + 1 mM GABA; (4) As 30 mg/kg + 0 mM GABA; (5) As 30 mg/kg + 0.5 mM GABA; (6) As 30 mg/kg + 1 mM GABA; (7) As 50 mg/kg + 0 mM GABA; (8) As 50 mg/kg + 0.5 mM GABA; (9) As 50 mg/kg + 1 mM GABA; (10) As 70 mg/kg + 0 mM GABA; (11) As 70 mg/kg + 0.5 mM GABA; and (12) As 70 mg/kg + 1 mM GABA.

An equal dose of NPK fertilizer (19:19:19) from ammonium nitrate, triple superphosphate and potassium sulfate was added to the media two times (5 g/pot). The first application occurred two weeks after the seedlings were transplanted, and the second application occurred one month later. Arsenic (As) was added after two weeks of transplanting to the soil at concentrations of 30, 50, or 70 mg/kg of soil. After another two weeks, GABA was sprayed foliarly for the first time at concentrations of 0.5 or 1 mM (which is sprayed as a preventative dose before the appearance of arsenic toxicity symptoms in plants), while the control plants were sprayed with distilled water. Each plant sprayed up to the saturation point, i.e., until the first drop. Two drops per liter of tween-20 was added to GABA solution as a surfactant. The plants were sprayed in the early morning. The last treatment was repeated one month after the 1 st spray (this coincided with the appearance of toxicity symptoms in plants to varying degrees). The plants were harvested at the end of the growing season, after the full opening of the inflorescences.

### Environmental conditions

The plants were grown during the summer season when the average temperature was 35 ± 5 °C during the day and 22 ± 2 °C at night, the average humidity was 22 ± 3%, and the hours of light were 14 and darkness 10.

### Chemicals used

Sodium arsenate salt (Na_2_HAsO^4^•7H_2_O) was used as the source of arsenic (As^5+^)^,^ and 30, 50, and 70 mg As^5^ kg^_1^ soil was mixed into the soil in the pots, as described by Liu et al. [10]. It was mixed with talcum powder, then scattered and mixed with the soil, followed by a light watering to ensure even distribution. Gamma‒butyric acid (GABA), which is soluble in water, was obtained from Bioworld Company in powder form. Its purity was 98%. It was prepared as a water solution. It was dissolved in distilled water.

### Data recorded

#### Vegetative characteristics

Plant height (cm) - stem diameter (cm) - number of branches/plant- number of leaves/plant - fresh weight of shoot/plant (g) - dry weight of shoot/plant (g) - fresh weight of roots/plant (g) - dry weight of roots/plant (g) – root length (cm) - leaf area (cm^2^).

#### Flowering characteristics

Number of spikes/plant - Spike part length (cm) - Fresh weight of spikes/plant (g) - Dry weight of spikes/plant (g).

#### Biochemical analysis

The total sugar content in fresh leaves was determined via the phenol‒sulfuric acid method, according to Dubois et al. [11]. The photosynthetic pigment content in fresh leaves (chlorophyll a, b and carotenoids mg/g F.W.) was evaluated according to Nornai [12]. The total anthocyanin content in fresh spikes (g/100 g F.W.) was determined according to Husia et al. [13]. The total proline content in fresh leaves was determined according to the method described by Bates et al. [14]. The total phenol content in fresh leaves (mg/g F.W.) was measured according to Quettier-Deleu et al. [15]. The total flavonoid content in fresh tepals was determined in absolute ethanolic extracts via aluminum chloride on the 8th day via the colorimetric method described by Chang et al. [16]. The arsenic content in the shoots, roots and soil was determined at Soil, Water and Environment Research Institute, Agricultural Research Center, Giza, Egypt.

#### Malondialdehyde (MDA) content and hydrogen peroxide (H_2_O_2_) content

To evaluate membrane lipid peroxidation, the MDA content was measured via the method of Zhang and Qu [17]. Powdered leaf tissue was mixed with a mixture of TCA and TBA after liquid nitrogen treatment, and the MDA content was measured. Finally, the absorption of the samples at 532 nm was read via a spectrophotometer (SCINCO, S-3100).

The amount of hydrogen peroxide (H_2_O_2_) in plant leaves was measured via a reaction with potassium iodide (KI) [18]. Leaves (0.2 g) were ground with 80% acetone and centrifuged, and the resulting extract was reacted with a mixture containing potassium phosphate and potassium iodide. The absorption of the samples at 390 nm was subsequently measured via a spectrophotometer (SCINCO, S-3100), and the H_2_O_2_ content was subsequently calculated on the basis of the standard curve (Tab. 1).

**Table 1:** The physical and chemical properties of the used soil

	Clay%	Silt%			Sand%				Texture	
Physical Properties	10.00	27.00			63.00				Sandy loam	
Chemical Properties	pH	EC (dS/m)								
				Soluble Cations (mmol/l)				Soluble anions (mmol/l)		
	8.00	4.91	Ca^++^	Mg^++^	Na^+^	K^+^	CO_3_^--^	HCO_3_^-^	Cl^-^	SO_4_^-^
			31.00	9.35	16.96	0.72	_	8.02	45.00	5.00
Macro and Micro nutrients (mg/kg)			N^+^	K^+^	P^---^	Cu^++^	Fe^++^	Mn^++^	Zn^++^	As^---^
			123.00	87.00	107.20	2.04	21.26	17.09	11.40	0.01

*dS/m* DeciSiemens per meter, *EC* Electrical conductivity

#### Superoxide dismutase (SOD) isoenzyme expression

Electrophoresis was performed to identify superoxide dismutase isoenzymes (SOD, E.C. 1.15.1.1) in 0.5 g fresh leaves as described by Winterbourn et al. [19]. Quantitative analysis of isoenzyme bands was performed using ImageJ software following background correction and lane normalization. Densitometric profiles demonstrated defined peaks corresponding to individual isoenzyme bands in each lane. Relative band intensity varied significantly among treatments [20].

### Statistical analysis

Data were analyzed by two-way analysis of variance (ANOVA). Then, Duncan’s New Multiple Range was used to determine the least significant difference test for comparison when significant differences were at *p* < 0.05 [21]. These analyses were performed using statistical analysis software COSTATV-63. Microsoft Excel Worksheet 2010 was utilized to figure the standard error.

## Results

### Vegetative growth

All the vegetative growth characteristics were negatively affected by the addition of As to the soil. Moreover, this effect significantly increased with increasing As concentration (Tables 2 and 3). In contrast, these characteristics were positively impacted by spraying GABA, and 1 mM GABA resulted in the highest values (Tables 2 and 3). With respect to the interaction effect on the celosia plants, spraying GABA on the polluted soil significantly enhanced all the vegetative growth features compared with those of the plants cultured in contaminated soil without the addition of GABA. Compared to 0.5 mM GABA, 1 mM GABA was more effective in both seasons.

**Table 2:** Some vegetative growth features affected by As and/or GABA during the 2023 and 2024 seasons

Plant Height (cm)								
1^st^ season					2^nd^ season			
	GABA (0 mM)	GABA (0.5 mM)	GABA (1 mM)	Mean	GABA (0 mM)	GABA (0.5 mM)	GABA (1 mM)	Mean
As (0 mg/kg)	61.00^ef^±0.58	68.08^ab^±0.70	69.27^a^±0.37	66.11^a^±0.17	61.27^de^±0.72	68.32^b^±0.42	71.33^a^±1.35	66.97^a^±0.48
As (30 mg/kg)	59.92^f^±0.83	62.67^de^±0.66	67.47^ab^±0.56	63.35^b^±0.14	60.46^e^±0.90	63.36^cd^±0.96	68.33^b^±0.88	64.05^b^±0.04
As (50 mg/kg)	53.77^h^±0.39	56.60^g^±1.06	65.87^bc^±0.77	58.74^c^±0.33	54.34^g^±0.69	56.83^f^±0.49	65.82^c^±0.74	59.00^c^±0.13
As (70 mg/kg)	49.40^i^±1.36	52.70^h^±0.85	64.90^cd^±0.87	55.67^d^±0.29	50.31^h^±0.69	53.83^g^±0.73	65.43^c^±1.12	56.52^d^±0.24
Mean	56.02^c^±0.36	60.01^b^±0.15	66.88^a^±0.19		56.59^c^±0.09	60.59^b^±0.21	67.73^a^±0.23	

Stem Diameter (cm)								
	1^st^ season				2^nd^ season			
	GABA (0 mM)	GABA (0.5 mM)	GABA (1 mM)	Mean	GABA (0 mM)	GABA (0.5 mM)	GABA (1 mM)	Mean
As (0 mg/kg)	1.61^c^±0.01	1.71^b^±0.01	1.96^a^±0.02	1.76^a^±0.01	1.62^c^±0.01	1.73^b^±0.01	1.99^a^±0.02	1.78^a^±0.00
As (30 mg/kg)	1.21^h^±0.01	1.36^f^±0.02	1.52^d^±0.01	1.36^b^±0.01	1.23^i^±0.01	1.37^g^±0.02	1.53^d^±0.01	1.37^b^±0.00
As (50 mg/kg)	1.17^h^±0.02	1.30^g^±0.01	1.46^e^±0.02	1.31^c^±0.00	1.19^i^±0.02	1.33^h^±0.01	1.48^e^±0.01	1.33^c^±0.00
As (70 mg/kg)	1.11^i^±0.02	1.28^g^±0.01	1.39^f^±0.01	1.26^d^±0.00	1.13^j^±0.01	1.32^h^±0.01	1.42^f^±0.01	1.29^d^±0.00
Mean	1.27^c^±0.00	1.41^b^±0.00	1.58^a^±0.00		1.29^c^±0.00	1.44^b^±0.00	1.60^a^±0.00	

NO. of branches/plant								
	1^st^ season				2^nd^ season			
	GABA (0 mM)	GABA (0.5 mM)	GABA (1 mM)	Mean	GABA (0 mM)	GABA (0.5 mM)	GABA (1 mM)	Mean
As (0 mg/kg)	29.00^bc^±0.58	30.00^ab^±1.15	32.00^a^±1.15	30.33^a^±0.33	30.33^ab^±0.88	30.67^ab^±1.20	33.00^a^±1.15	31.33^a^±0.17
As (30 mg/kg)	23.00^fg^±0.58	24.00^ef^±0.58	25.67^de^±0.88	24.22^b^±0.18	23.67^ef^±0.88	25.00^de^±0.58	26.67^cd^±0.88	25.11^b^±0.18
As (50 mg/kg)	21.00^g^±0.58	23.67^ef^±0.88	27.33^cd^±0.88	24.00^b^±0.18	21.67^f^±0.88	23.6^ef^±1.20	28.00^bc^±0.58	24.44^b^±0.31
As (70 mg/kg)	13.67^i^±0.88	16.67^h^±0.88	22.33^fg^±0.88	17.56^c^±0.00	14.00^h^±0.58	17.33^g^±0.88	23.00^ef^±1.15	18.11^c^±0.29
Mean	21.67^c^±0.13	23.58^b^±0.20	26.83^a^±0.12		22.42^c^±0.13	24.17^b^±0.26	27.67^a^±0.24	

NO. of leaves/plant								
	1^st^ season				2^nd^ season			
	GABA (0 mM)	GABA (0.5 mM)	GABA (1 mM)	Mean	GABA (0 mM)	GABA (0.5 mM)	GABA (1 mM)	Mean
As (0 mg/kg)	223.00^c^±1.15	235.00^b^±0.58	256.33^a^±2.03	238.11^a^±0.73	223.33^c^±2.73	236.00^b^±1.15	258.67^a^±0.88	239.33^a^±1.00
As (30 mg/kg)	191.00^e^±0.58	193.00^e^±0.58	217.67^d^±1.45	200.56^b^±0.51	192.00^e^±1.15	193.67^e^±0.67	219.00^cd^±1.53	201.56^b^±0.43
As (50 mg/kg)	185.67^f^±1.45	190.33^e^±0.58	215.33^d^±1.20	197.11^c^±0.29	187.00^f^±1.53	191.33^ef^±0.88	216.33^d^±1.20	198.22^c^±0.32
As (70 mg/kg)	158.67^i^±1.76	169.33^h^±2.03	174.00^g^±1.53	167.33^d^±0.25	159.67^i^±1.76	170.67^h^±1.76	175.33^g^±1.45	168.56^d^±0.18
Mean	189.58^c^±0.44	196.92^b^±0.60	215.83^a^±0.30		190.50^c^±0.58	197.92^b^±0.41	217.33^a^±0.25	

Root Length (cm)								
	1^st^ season				2^nd^ season			
	GABA (0 mM)	GABA (0.5 mM)	GABA (1 mM)	Mean	GABA (0 mM)	GABA (0.5 mM)	GABA (1 mM)	Mean
As (0 mg/kg)	39.33^b^±1.76	40.67^ab^±1.45	44.67^a^±1.20	41.56^a^±0.28	40.33^bc^±0.45	42.00^ab^±0.15	46.00^a^±0.15	42.78^a^±0.17
As (30 mg/kg)	28.67^de^±1.45	33.33^cd^±1.76	36.67^bc^±1.76	32.89^b^±0.18	30.00e±1.15	34.33^de^±1.20	38.00^bcd^±2.08	34.11^b^±0.52
As (50 mg/kg)	24.67^ef^±1.45	30.33^d^±1.45	36.00^bc^±2.31	30.33^bc^±0.48	25.33^f^±2.40	31.33^e^±1.45	37.00^cd^±2.08	31.22^c^±0.48
As (70 mg/kg)	22.00^f^±1.53	28.67^de^±1.76	33.33^cd^±0.88	28.00^c^±0.46	23.33^f^±1.76	30.33^e^±0.88	34.00^de^±1.15	29.22^c^±0.45
Mean	28.67^c^±0.13	33.25^b^±0.16	37.67^a^±0.55		29.75^c^±0.46	34.50^b^±0.20	38.75^a^±0.46	

Means between treatments in the same column followed by the same letter were not significantly different according to Duncan’s multiple range test (DMRT) at p < 0.05

**Table 3:** Some vegetative growth features affected by As and/or GABA during the 2023 and 2024 seasons

Shoot F.W. (g)								
1^st^ season					2^nd^ season			
	GABA (0 mM)	GABA (0.5 mM)	GABA (1 mM)	Mean	GABA (0 mM)	GABA (0.5 mM)	GABA (1 mM)	Mean
As (0 mg/kg)	134.41^bc^±0.32	137.93^ab^±1.08	141.60^a^±2.13	137.98^a^±0.91	135.32^b^±1.06	138.40^b^±1.32	143.58^a^±1.50	139.10^a^±0.22
As (30 mg/kg)	92.90^e^±1.28	106.34^d^±0.88	133.29^c^±2.09	110.84^b^±0.62	93.75^d^±0.79	107.25^c^±2.50	134.01^b^±2.04	111.67^b^±0.88
As (50 mg/kg)	65.25^h^±1.44	92.53^e^±1.18	103.47^d^±0.69	87.08^c^±0.38	66.65^g^±1.67	93.56^d^±1.28	104.14^c^±0.65	88.12^c^±0.51
As (70 mg/kg)	60.52^i^±2.44	75.30^g^±1.34	86.77^f^±0.60	74.20^d^±0.93	61.45^h^±2.44	76.37^f^±0.90	87.36^e^±0.42	75.06^d^±1.06
Mean	88.27^c^±0.75	103.02^b^±0.17	116.28^a^±0.74		89.29^c^±0.63	103.89^b^±0.60	117.28^a^±0.65	

Shoot D.W. (g)								
	1^st^ season				2^nd^ season			
	GABA (0 mM)	GABA (0.5 mM)	GABA (1 mM)	Mean	GABA (0 mM)	GABA (0.5 mM)	GABA (1 mM)	Mean
As (0 mg/kg)	14.44^b^±0.49	17.88^a^±0.56	18.36^a^±0.90	16.90^a^±0.22	15.47^b^±0.44	18.63^a^±0.35	19.32^a^±1.13	17.81^a^±0.43
As (30 mg/kg)	12.65^b^±0.96	13.73^b^±0.88	14.04^b^±0.79	13.47^b^±0.08	13.56^b^±0.85	14.53^b^±1.49	15.28^b^±0.78	14.46^b^±0.39
As (50 mg/kg)	8.65^c^±1.04	12.32^b^±0.80	13.15^b^±0.61	11.37^c^±0.21	9.83^c^±1.20	13.73^b^±0.93	14.65^b^±0.36	12.74^c^±0.43
As (70 mg/kg)	6.22^d^±1.10	7.58^cd^±0.28	9.08^c^±0.69	7.63^d^±0.41	7.03^d^±1.50	8.21^cd^±0.50	10.34^c^±0.34	8.53^d^±0.63
Mean	10.49^b^±0.24	12.88^a^±0.23	13.66^a^±0.11		11.48^b^±0.40	13.78^a^±0.44	14.90^a^±0.33	

Root F.W. (g)								
	1^st^ season				2^nd^ season			
	GABA (0 mM)	GABA (0.5 mM)	GABA (1 mM)	Mean	GABA (0 mM)	GABA (0.5 mM)	GABA (1 mM)	Mean
As (0 mg/kg)	18.15^ab^±1.35	18.60^ab^±0.39	21.67^a^±2.17	19.48^a^±0.89	19.28^ab^±1.65	19.82^ab^±0.63	22.34^a^±2.04	20.48^a^±0.73
As (30 mg/kg)	14.86^bc^±1.42	15.73^bc^±1.06	17.43^bc^±1.84	16.01^b^±0.39	15.67^cd^±1.42	16.77^bcd^±1.03	18.76^abcd^±1.74	17.07^b^±0.35
As (50 mg/kg)	9.63^d^±1.28	10.09^d^±1.41	14.00^c^±1.44	11.24^c^±0.09	10.31^e^±1.10	11.18^e^±1.39	15.01^d^±1.42	12.17^c^±0.17
As (70 mg/kg)	7.00^d^±1.10	8.00^d^±0.10	9.40^d^±0.33	8.13^d^±0.52	8.11^e^±1.19	9.15^e^±0.49	10.32^e^±0.44	9.19^d^±0.42
Mean	12.41^b^±0.12	13.11^b^±0.52	15.63^a^±0.70		13.34^b^±0.21	14.23^b^±0.35	16.61^a^±0.60	

Root D.W. (g)								
	1^st^ season				2^nd^ season			
	GABA (0 mM)	GABA (0.5 mM)	GABA (1 mM)	Mean	GABA (0 mM)	GABA (0.5 mM)	GABA (1 mM)	Mean
As (0 mg/kg)	5.41^ab^±0.23	5.59^ab^±0.95	6.69^a^±0.60	5.90^a^±0.36	5.99^ab^±0.96	6.78^ab^±1.02	7.18^a^±0.59	6.65^a^±0.24
As (30 mg/kg)	3.48^bc^±0.93	3.97^bc^±0.57	4.98^bc^±0.46	4.14^b^±0.24	4.74^cd^±0.65	4.55^bcd^±0.51	5.92^abcd^±0.11	5.07^b^±0.28
As (50 mg/kg)	1.48^d^±0.30	1.68^d^±0.07	2.93^c^±0.33	2.03^c^±0.14	1.84^e^±0.17	1.99^e^±0.11	3.63^d^±0.32	2.49^c^±0.11
As (70 mg/kg)	1.38^d^±0.19	1.44^d^±0.09	1.69^d^±0.25	1.50^c^±0.08	1.88^e^±0.06	1.95^e^±0.06	2.31^e^±0.21	2.05^c^±0.09
Mean	2.94^b^±0.30	3.17^b^±0.36	4.07^a^±0.08		3.61^b^±0.36	3.82^b^±0.39	4.76^a^±0.17	

Leaf Area (cm^2^)								
	1^st^ season				2^nd^ season			
	GABA (0 mM)	GABA (0.5 mM)	GABA (1 mM)	Mean	GABA (0 mM)	GABA (0.5 mM)	GABA (1 mM)	Mean
As (0 mg/kg)	8.00^bc^±0.50	8.50^ab^±0.29	9.17^a^±0.73	8.56^a^±0.22	8.50^bc^±0.29	8.83^ab^±0.17	9.50^a^±0.29	8.94^a^±0.07
As (30 mg/kg)	7.17^cde^±0.33	7.67^bcd^±0.17	7.83^bcd^±0.44	7.56^b^±0.14	7.50^de^±0.29	7.83^cde^±0.17	8.17^bcd^±0.33	7.83^b^±0.09
As (50 mg/kg)	6.17^ef^±0.17	6.83^de^±0.17	7.83^bcd^±0.33	6.94^c^±0.10	6.33^gh^±0.17	7.17^ef^±0.17	8.00^cd^±0.29	7.17^c^±0.07
As (70 mg/kg)	4.00^fg^±0.29	5.33^f^±0.44	6.33^ef^±0.44	5.22^d^±0.09	4.33^i^±0.17	5.67^h^±0.44	6.67^fg^±0.17	5.56^d^±0.16
Mean	6.33^c^±0.12	7.08^b^±0.11	7.79^a^±0.15		6.67^c^±0.06	7.38^b^±0.12	8.08^a^±0.06	

### Flowering characteristics

Although arsenic (As) negatively affected the number of flowers per plant compared to the control, the overall number of flowers increased with higher concentrations of As. The 1 mM GABA treatment resulted in the greatest amount of flowers/plant (Table 4). For the interaction effect, 1 mM GABA alone had the greatest effect (127.33 ± 2.40 and 130.33 ± 1.45 flowers/plant in the 1 st and 2nd seasons, respectively) compared to the control (91.67 ± 2.03 and 93.33 ± 1.76 flowers/plant in the 1 st and 2nd seasons, respectively). Moreover, 1 mM GABA effectively enhanced this feature in the polluted soils, even at high concentrations, as spraying 1 mM GABA on plants cultured in soils polluted with 70 ml of As resulted in a greater number of flowers/plant than in the control (104.33 ± 1.76 and 106.00 ± 1.73 flowers/plant in the 1 st and 2nd seasons, respectively) (Table 4).

**Table 4:** Flowering characteristics affected by As and/or GABA during the 2023 and 2024 seasons

NO. of flowers/plant								
1^st^ season					2^nd^ season			
	GABA (0 mM)	GABA (0.5 mM)	GABA (1 mM)	Mean	GABA (0 mM)	GABA (0.5 mM)	GABA (1 mM)	Mean
As (0 mg/kg)	91.67d±2.03	112.67b±1.76	127.33a±2.40	110.56a±0.32	93.33d±1.76	114.00b±2.08	130.33a±1.45	112.56a±0.31
As (30 mg/kg)	42.33h±1.45	56.67g±1.76	71.00f±1.15	56.67d±0.30	43.33i±1.45	57.67h±1.76	72.00f±1.15	57.67d±0.30
As (50 mg/kg)	44.00h±1.53	62.33g±1.20	85.00e±1.15	63.78c±0.20	46.00i±1.15	64.33g±1.45	87.33e±1.76	65.89c±0.30
As (70 mg/kg)	58.67g±1.76	72.67f±1.45	104.33c±1.76	78.56b±0.18	60.67gh±1.20	74.33f±1.45	106.00c±1.73	80.33b±0.27
Mean	59.17c±0.22	76.08b±0.24	96.92a±0.52		60.83c±0.24	77.58b±0.26	98.92a±0.25	

Flowers F.W. (g)								
	1^st^ season				2^nd^ season			
	GABA (0 mM)	GABA (0.5 mM)	GABA (1 mM)	Mean	GABA (0 mM)	GABA (0.5 mM)	GABA (1 mM)	Mean
As (0 mg/kg)	22.67a±1.91	23.77a±0.92	25.47a±1.65	23.97a±0.52	24.06a±1.68	25.39a±0.89	27.28a±1.49	25.58a±0.41
As (30 mg/kg)	13.24bc±1.00	16.03b±0.89	22.23a±1.20	17.17b±0.16	14.53bc±0.97	17.15b±0.95	23.28a±1.09	18.32b±0.08
As (50 mg/kg)	11.27bcd±1.64	14.84b±1.57	16.15b±0.03	14.09c±0.91	12.44bcd±1.38	15.89b±1.67	16.84b±0.15	15.06c±0.80
As (70 mg/kg)	7.84d±0.63	9.03cd±0.90	11.23bcd±1.12	9.37d±0.24	8.53d±0.50	10.25cd±1.11	12.33bcd±1.13	10.37d±0.36
Mean	13.76c±0.51	15.92b±0.29	18.77a±0.59		14.89c±0.44	17.17b±0.31	19.93a±0.49	

Flowers DW. (g)								
	1^st^ season				2^nd^ season			
	GABA (0 mM)	GABA (0.5 mM)	GABA (1 mM)	Mean	GABA (0 mM)	GABA (0.5 mM)	GABA (1 mM)	Mean
As (0 mg/kg)	5.20ab±0.62	6.05ab±0.56	6.54a±0.33	5.93a±0.16	6.32ab±0.61	7.24a±0.53	7.46a±0.56	7.00a±0.04
As (30 mg/kg)	2.69d±0.12	3.60cd±0.68	4.78bc±0.78	3.69b±0.35	3.11d±0.05	4.51cd±0.42	5.62bc±0.49	4.41b±0.24
As (50 mg/kg)	1.29e±0.20	2.87d±0.14	3.48cd±0.17	2.55c±0.03	1.54e±0.31	3.22d±0.21	4.01d±0.07	2.92c±0.12
As (70 mg/kg)	0.72e±0.33	0.87e±0.24	1.02e±0.12	0.87d±0.11	1.05e±0.29	1.10e±0.39	1.12e±0.12	1.09d±0.14
Mean	2.48b±0.19	3.35a±0.22	3.95a±0.26		3.00b±0.20	4.02a±0.12	4.55a±0.22	

Means between treatments in the same column followed by the same letter were not significantly different according to Duncan’s multiple range test (DMRT) at p < 0.05

**Fig. 1:**
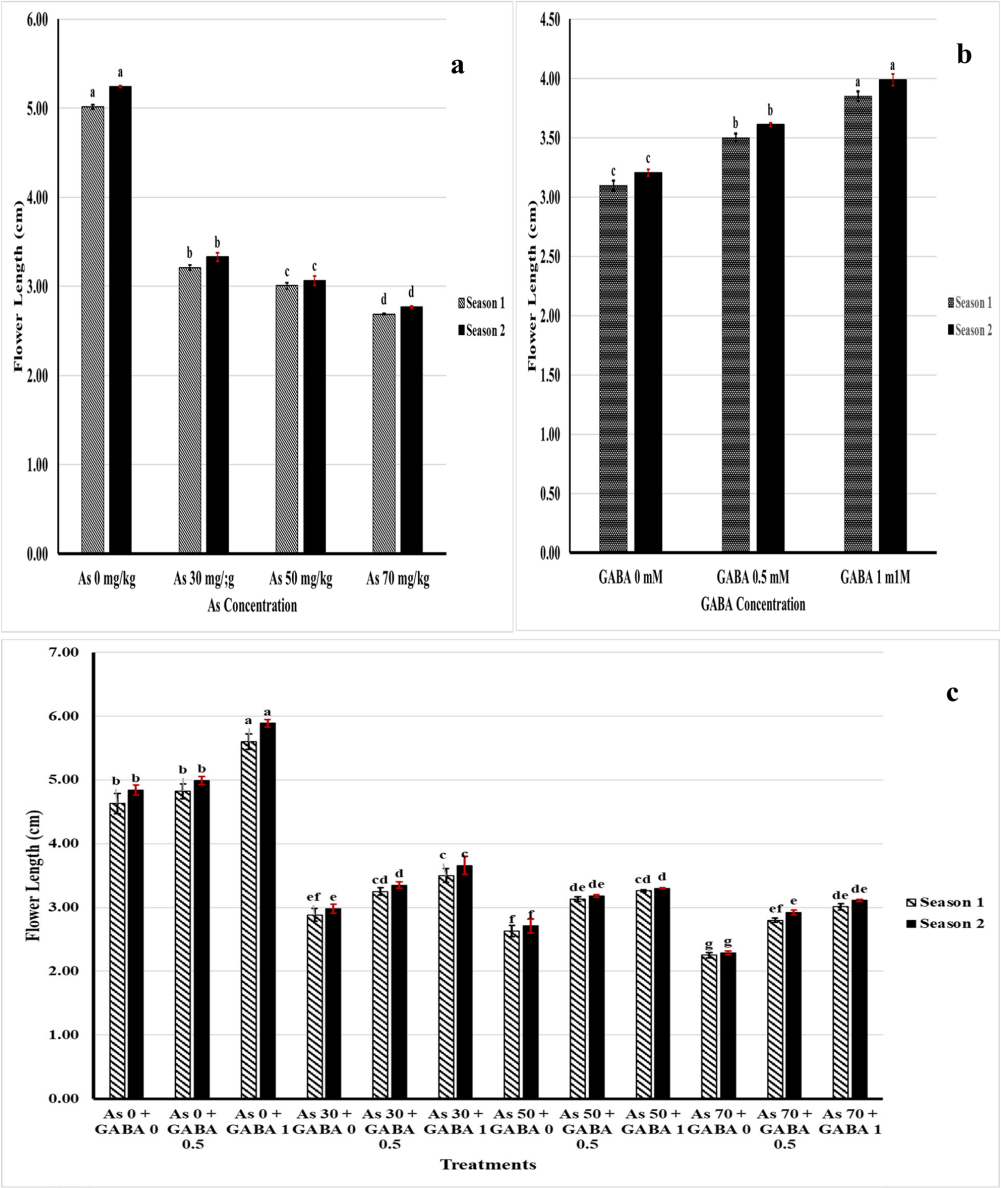
Flower length (cm) affected by As (**a**), GABA (**b**), and As + GABA (**c**) during the 2023 and 2024 seasons

The length of celosia flowers decreased with increasing concentrations of As. In contrast, as the GABA concentration increased, the length of the flower increased. In terms of the interaction effect, the plants treated with 1 mM GABA presented the highest values in both seasons. GABA at the two applied concentrations prolonged the length of celosia flowers in the soils contaminated with different concentrations of As, and 1 mM GABA had the greatest effect (Fig. 1).

The data in Table 4 indicate that As negatively affected the fresh and dry weights of the flowers; however, GABA positively affected the fresh and dry weights of the flowers, as there were no significant differences in dry weight between the two concentrations of GABA. There were no significant differences in fresh weight or dry weight between the control and the two concentrations of GABA. GABA increased the fresh and dry weights of the flowers in the As-polluted soils. However, the two concentrations did not significantly affect the dry weight of the flowers at high concentrations of As.

**Fig. 2:**
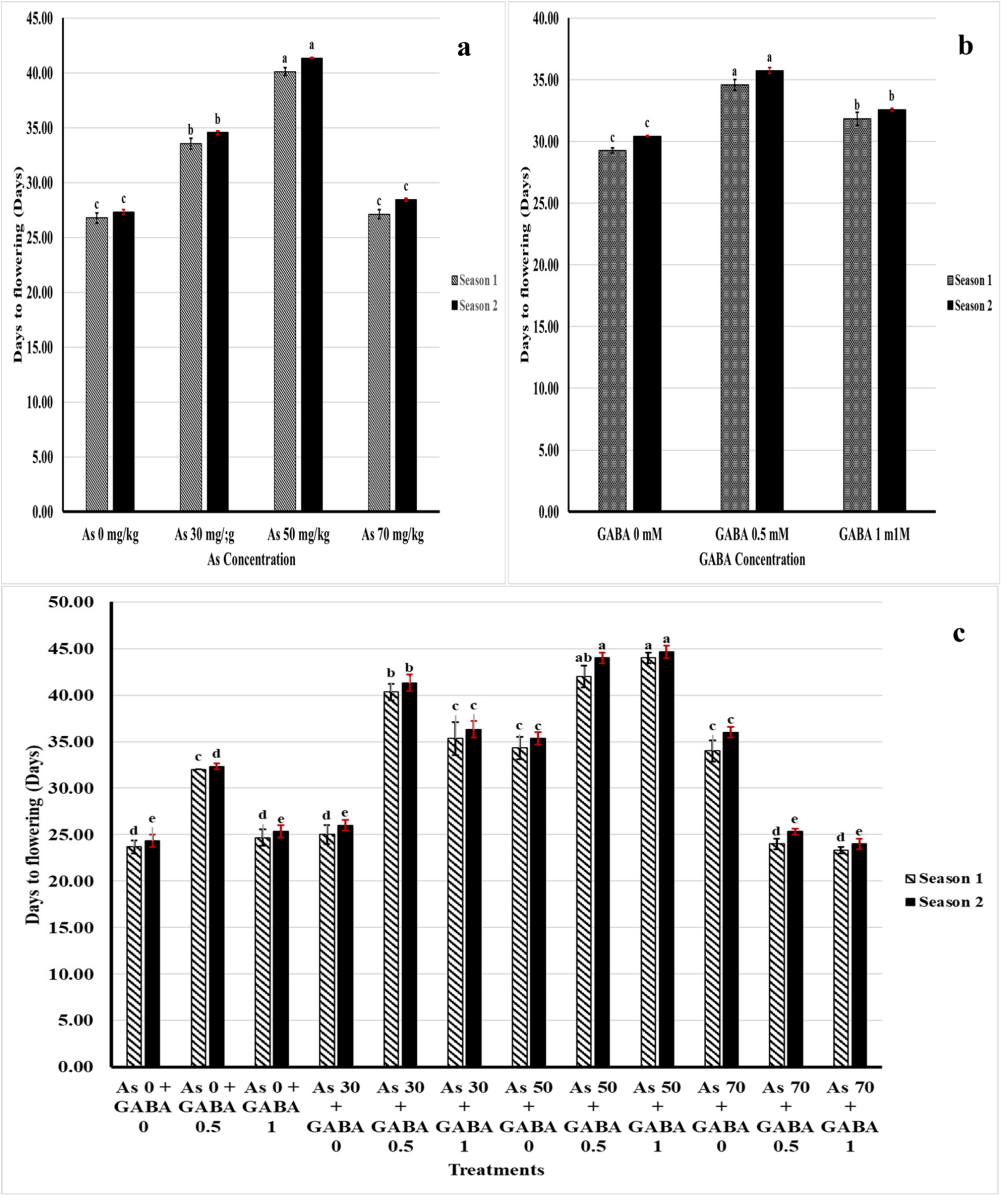
Days to flowering affected by As (**a**), GABA (**b**), and As + GABA (**c**) during the 2023 and 2024 seasons

Arsenic at concentrations of 30 and 50 mg caused a delay in flowering compared with the control plants. The concentration of 50 mg had the greatest influence, as it delayed flowering for approximately 14 days after the control plants did (Fig. 2). On the other hand, the two concentrations of GABA significantly delayed flowering compared to the control, and 0.5 mM GABA resulted in the greatest delay in the two seasons. In terms of the interaction effect, 23.67 ± 0.67 and 24.33 ± 0.67 days were recorded for the control plants in the 1 st and 2nd seasons, respectively, whereas 44.00 ± 0.58 and 44.67 ± 0.67 ± 0.67 days were registered for the As (50 mg/kg) + GABA 1 mM treatment in the 1 st and 2nd seasons, respectively; i.e., this treatment delayed flowering for approximately 20 days in both seasons.

### Biochemical analysis

#### Photosynthetic pigments (mgg^−1^ of fresh leaves)

While As at 30 mg/kg significantly decreased the amount of chlorophyll a and total carotenoids in the leaves compared to those in the control, As at 50 and 70 mg/kg significantly increased these photosynthetic pigments in the leaves compared to the control, and 50 mg/kg As had the highest values (Table 5). Compared to the control, all the applied concentrations of As significantly increased chlorophyll b, with a greater effect at a concentration of 70 mg/kg. Similarly, compared to the control, GABA at the two applied concentrations dramatically increased the amount of chlorophyll a, b, and total carotenoids. The 0.5 mM GABA treatment had the highest values in the two seasons. For the interaction effect, As (50 mg/kg) + GABA (0.5 mM) had the highest values of chlorophyll a and total carotenoid content, whereas As (70 mg/kg) alone caused the highest values of chlorophyll b (Table 5).

**Table 5:** Photosynthetic pigments of leaves and anthocyanin pigments of flowers affected by As and/or GABA during the 2023 and 2024 seasons

Chlorophyll a (mgg^-1^ of F.W. leaves)								
1^st^ season					2^nd^ season			
	GABA (0 mM)	GABA (0.5 mM)	GABA (1 mM)	Mean	GABA (0 mM)	GABA (0.5 mM)	GABA (1 mM)	Mean
As (0 mg/kg)	0.410j±0.002	0.589g±0.005	0.764d±0.002	0.588c±0.002	0.412k±0.000	0.597g±0.001	0.775d±0.001	0.595c±0.000
As (30 mg/kg)	0.383k±0.002	0.516i±0.013	0.629f±0.002	0.509d±0.006	0.389l±0.001	0.541j±0.001	0.637f±0.001	0.522d±0.000
As (50 mg/kg)	0.554h±0.002	1.230a±0.002	0.815c±0.003	0.866a±0.000	0.564i±0.001	1.236a±0.000	0.834c±0.001	0.878a±0.000
As (70 mg/kg)	1.009b±0.003	0.663e±0.003	0.566h±0.002	0.746b±0.001	1.019b±0.000	0.697e±0.001	0.583h±0.002	0.766b±0.001
Mean	0.589c±0.000	0.749a±0.004	0.693b±0.000		0.596c±0.000	0.768a±0.000	0.707b±0.000	

Chlorophyll b (mgg^-1^ of F.W. leaves)								
	1^st^ season				2^nd^ season			
	GABA (0 mM)	GABA (0.5 mM)	GABA (1 mM)	Mean	GABA (0 mM)	GABA (0.5 mM)	GABA (1 mM)	Mean
As (0 mg/kg)	0.045l±0.001	0.067i±0.002	0.074h±0.002	0.062d±0.001	0.053i±0.001	0.079g±0.001	0.088f±0.001	0.073d±0.000
As (30 mg/kg)	0.051k±0.002	0.101g±0.000	0.104f±0.003	0.085c±0.001	0.064h±0.002	0.122e±0.001	0.124e±0.001	0.103c±0.001
As (50 mg/kg)	0.058j±0.001	0.136d±0.001	0.127e±0.001	0.107b±0.000	0.076g±0.001	0.140c±0.001	0.129d±0.001	0.115b±0.001
As (70 mg/kg)	0.259a±0.002	0.201b±0.001	0.192c±0.001	0.217a±0.001	0.281a±0.002	0.208b±0.002	0.205b±0.001	0.231a±0.000
Mean	0.103b±0.001	0.126a±0.001	0.124a±0.001		0.119b±0.000	0.137a±0.001	0.136a±0.000	

Total carotenoid content (mgg^-1^ of F.W. leaves)								
	1^st^ season				2^nd^ season			
	GABA (0 mM)	GABA (0.5 mM)	GABA (1 mM)	Mean	GABA (0 mM)	GABA (0.5 mM)	GABA (1 mM)	Mean
As (0 mg/kg)	0.288k±0.002	0.415f±0.002	0.586d±0.002	0.429c±0.000	0.312k±0.002	0.438f±0.002	0.605c±0.000	0.452c±0.001
As (30 mg/kg)	0.281l±0.001	0.386h±0.002	0.427e±0.004	0.365d±0.001	0.286l±0.000	0.398h±0.001	0.476e±0.001	0.386d±0.001
As (50 mg/kg)	0.409g±0.002	0.851a±0.001	0.594c±0.001	0.618a±0.000	0.432g±0.001	0.856a±0.000	0.599d±0.001	0.629a±0.000
As (70 mg/kg)	0.725b±0.001	0.358i±0.002	0.349j±0.001	0.477b±0.000	0.728b±0.000	0.364i±0.000	0.351j±0.000	0.481b±0.000
Mean	0.426c±0.000	0.502a±0.000	0.489b±0.001		0.439c±0.001	0.514a±0.001	0.508b±0.000	

Total anthocyanin content (g/100 g of F.W. petals)								
	1^st^ season				2^nd^ season			
	GABA (0 mM)	GABA (0.5 mM)	GABA (1 mM)	Mean	GABA (0 mM)	GABA (0.5 mM)	GABA (1 mM)	Mean
As (0 mg/kg)	0.035a±0.000	0.035a±0.001	0.037a±0.001	0.036a±0.000	0.036b±0.000	0.038b±0.001	0.040a±0.000	0.038a±0.000
As (30 mg/kg)	0.024d±0.001	0.031b±0.001	0.034a±0.001	0.030b±0.000	0.025g±0.001	0.033c±0.001	0.036b±0.001	0.031b±0.000
As (50 mg/kg)	0.018e±0.000	0.024d±0.001	0.025cd±0.002	0.023c±0.001	0.019h±0.000	0.026fg±0.001	0.027ef±0.001	0.024c±0.000
As (70 mg/kg)	0.014f±0.000	0.028c±0.000	0.028c±0.000	0.023c±0.000	0.015i±0.001	0.029de±0.000	0.030d±0.001	0.025c±0.000
Mean	0.023c±0.000	0.030b±0.000	0.031a±0.001		0.024c±0.000	0.031b±0.000	0.033a±0.000	

Means between treatments in the same column followed by the same letter were not significantly different according to Duncan’s multiple range test (DMRT) at p < 0.05

#### Total anthocyanin content (g/100 g of fresh petals)

The amount of anthocyanin pigments in celosia petals was negatively affected by soil polluted with As. In contrast, it was not affected by spraying GABA (Table 5). Moreover, GABA could ameliorate the negative effects of As on the total anthocyanin content. Spraying 1 mM GABA to plants cultured in contaminated soil with 30 mg/kg As increased the total anthocyanin content to a level close to that of the control in both seasons.

#### Total flavonoid content (mgg^−1^ of fresh leaves)

The data shown in Table (6) reveal that, compared to the control, As at concentrations of 30 and 50 mg/kg lowered the flavonoid content, whereas As at the concentration of 70 mg/kg caused a significant increase in the total flavonoid content compared to the control. In addition, the contents of flavonoids in the leaves of the plants treated with GABA at the two applied concentrations were greater than those in the control plants during the two seasons. With respect to the effect of GABA on As-polluted soils, the total flavonoid content increased as the concentration of GABA increased. Moreover, spraying GABA on plants cultured in polluted soils with As at 30 and 50 mg/kg increased the total flavonoid content compared to that of the plants that were not sprayed with GABA. However, 70 mg/kg As had the highest total flavonoid content compared to the control and the other treatments, and spraying GABA had a negative effect on the flavonoid content at this high concentration of As.

**Table 6:** Some biochemical components of leaves affected by As and/or GABA during the 2023 and 2024 seasons

Total flavonoid content (mgg^-1^ of F.W. leaves)								
1^st^ season					2^nd^ season			
	GABA (0 mM)	GABA (0.5 mM)	GABA (1 mM)	Mean	GABA (0 mM)	GABA (0.5 mM)	GABA (1 mM)	Mean
As (0 mg/kg)	1.03e±0.04	1.14d±0.03	1.25c±0.02	1.14b±0.01	1.07e±0.02	1.19d±0.01	1.26c±0.01	1.18b±0.01
As (30 mg/kg)	0.82h±0.02	0.86gh±0.03	0.97ef±0.04	0.88d±0.01	0.83g±0.02	0.89g±0.01	0.99f±0.04	0.90d±0.02
As (50 mg/kg)	0.88fgh±0.01	1.43b±0.03	0.94efg±0.03	1.09c±0.01	0.89g±0.01	1.46b±0.02	0.98f±0.02	1.11c±0.01
As (70 mg/kg)	1.63a±0.02	1.22c±0.03	0.98ef±0.02	1.28a±0.01	1.68a±0.02	1.25c±0.01	0.99f±0.02	1.31a±0.01
Mean	1.09b±0.01	1.16a±0.00	1.03c±0.01		1.12b±0.00	1.20a±0.01	1.06c±0.01	

Total phenol content (mgg^-1^ of F.W. leaves)								
	1^st^ season				2^nd^ season			
	GABA (0 mM)	GABA (0.5 mM)	GABA (1 mM)	Mean	GABA (0 mM)	GABA (0.5 mM)	GABA (1 mM)	Mean
As (0 mg/kg)	0.19de±0.00	0.16ef±0.01	0.11g±0.01	0.15c±0.00	0.18e±0.00	0.14f±0.01	0.10g±0.01	0.14d±0.00
As (30 mg/kg)	0.34b±0.01	0.25c±0.01	0.21d±0.01	0.27b±0.00	0.36b±0.00	0.24c±0.01	0.20d±0.01	0.27c±0.00
As (50 mg/kg)	0.49a±0.01	0.28c±0.01	0.13fg±0.01	0.30a±0.00	0.51a±0.01	0.25c±0.01	0.11g±0.01	0.29b±0.00
As (70 mg/kg)	0.50a±0.01	0.28c±0.01	0.16ef±0.01	0.32a±0.00	0.52a±0.00	0.26c±0.01	0.15f±0.01	0.31a±0.00
Mean	0.38a±0.00	0.24b±0.00	0.15c±0.00		0.39a±0.00	0.22b±0.00	0.14c±0.00	

Total proline content (mgg^-1^ of F.W. leaves)								
	1^st^ season				2^nd^ season			
	GABA (0 mM)	GABA (0.5 mM)	GABA (1 mM)	Mean	GABA (0 mM)	GABA (0.5 mM)	GABA (1 mM)	Mean
As (0 mg/kg)	0.34f±0.02	0.30f±0.01	0.27g±0.01	0.30d±0.00	0.32e±0.01	0.29f±0.00	0.25g±0.00	0.29d±0.00
As (30 mg/kg)	0.39e±0.01	0.34f±0.01	0.33f±0.01	0.36c±0.00	0.41d±0.00	0.33e±0.00	0.32e±0.01	0.35c±0.00
As (50 mg/kg)	0.61a±0.02	0.45c±0.01	0.41de±0.01	0.49b±0.01	0.63a±0.01	0.44c±0.00	0.40d±0.00	0.49b±0.00
As (70 mg/kg)	0.63a±0.01	0.54b±0.00	0.44cd±0.01	0.54a±0.00	0.64a±0.01	0.53b±0.00	0.43c±0.00	0.53a±0.00
Mean	0.49a±0.00	0.41b±0.00	0.36c±0.00		0.50a±0.00	0.40b±0.00	0.35c±0.00	

Total sugar content (% of F.W. leaves)								
	1^st^ season				2^nd^ season			
	GABA (0 mM)	GABA (0.5 mM)	GABA (1 mM)	Mean	GABA (0 mM)	GABA (0.5 mM)	GABA (1 mM)	Mean
As (0 mg/kg)	5.19i±0.01	5.27i±0.00	6.27h±0.02	5.58d±0.01	5.36i±0.01	5.47i±0.01	6.41h±0.02	5.75d±0.01
As (30 mg/kg)	7.91g±0.02	8.23fg±0.00	8.70f±0.00	8.28c±0.01	8.06g±0.02	8.26g±0.00	8.76f±0.00	8.36c±0.01
As (50 mg/kg)	11.83e±0.03	12.39d±0.03	13.21c±0.04	12.47b±0.01	11.91e±0.03	12.67d±0.03	13.47c±0.01	12.68b±0.01
As (70 mg/kg)	13.94b±0.00	14.07b±0.01	14.62a±0.02	14.21a±0.01	14.07b±0.00	14.28b±0.00	15.02a±0.01	14.46a±0.00
Mean	9.72c±0.01	9.99b±0.01	10.70a±0.01		9.85c±0.01	10.17b±0.01	10.91a±0.01	

Means between treatments in the same column followed by the same letter were not significantly different according to Duncan’s multiple range test (DMRT) at p < 0.05

#### Total phenol content (mgg^−1^ of fresh leaves)

The leaf content of phenols significantly exceeded in the As-treated plants at all the applied concentrations compared to the control plants, with As at 70 mg/kg being superior to the control plants (Table 6). In contrast, the phenol content dramatically decreased as the GABA concentration increased. These effects were clearly mirrored when GABA was applied to soils polluted with As. Compared to the control, As alone considerably increased the phenol content, whereas the addition of GABA resulted in a decrease in the phenol content compared to that of the plants that were not sprayed with GABA.

#### Total proline content (mgg^−1^ of fresh leaves)

The proline content was affected in the same way as the phenol content was. GABA alone or with As resulted in a decrease in the total proline content, whereas As alone resulted in an increase in the total proline content.

#### Total sugar content (% of fresh water)

Compared to the control, both As alone and GABA alone increased the percentage of sugar content. Furthermore, spraying GABA on plants subjected to As pollution dramatically increased the leaf sugar content. The highest values were obtained by applying As (70 mg/kg) + GABA (1 mM) (Table 5).

#### As contents in shoots, roots and soil

**Fig. 3:**
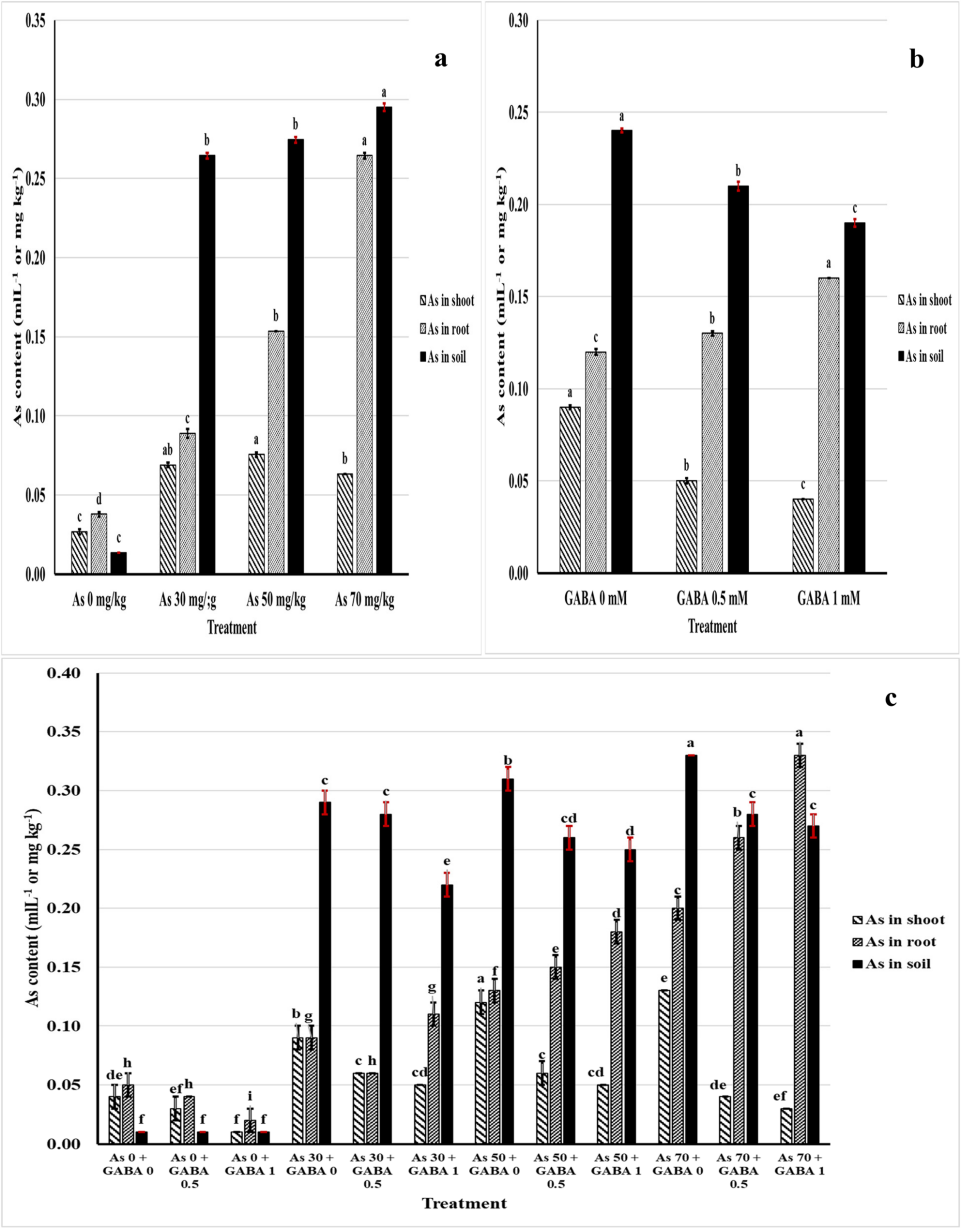
As contents in shoots, roots, and soil affected by As (**a**), GABA (**b**), and As + GABA during the 2024 season

GABA effectively reduced As accumulation in shoots and was highly efficacious at high concentrations of As, as 1 mM GABA with 70 mg/kg As resulted in values close to those of the control plants (Fig. 3). In contrast, GABA caused the accumulation of As in the roots, as the As content in the roots increased with increasing concentrations of GABA alone or with different concentrations of As. Thus, As (70 mg/kg) + GABA (1 mM) resulted in the highest values compared with those of the control. The As content of the soil decreased when GABA was utilized. GABA also slightly decreased the As content in contaminated soils, even at high concentrations.

### Malondialdehyde (MDA) and hydrogen peroxidase (H_2_O_2_) as markers of oxidative stress

**Fig. 4:**
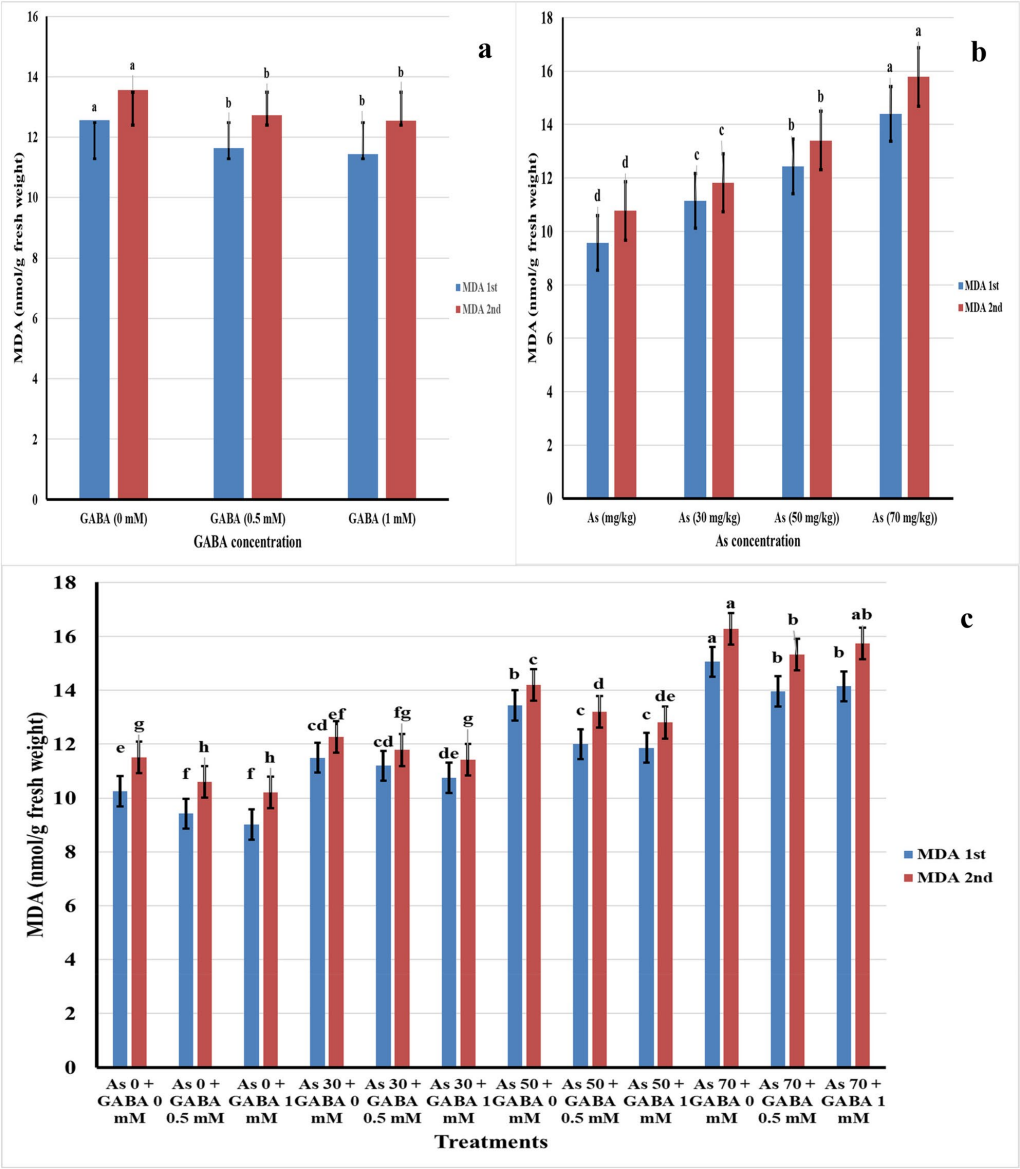
Effect of Arsenic Stress (**a**), GABA (**b**), and Their Interaction (**c**) on MDA content during the 2023 and 2024 seasons

As shown in Fig. 4, oxidative stress markers clearly reflect arsenic-induced cellular damage, as evidenced by changes in malondialdehyde (MDA) content. The MDA levels increased significantly by 33.5% and 31.7% during the first and second seasons, respectively, at 70 mg kg⁻¹ soil arsenic compared with the control. This increase indicates enhanced lipid peroxidation and membrane damage under arsenic stress.

When GABA was applied individually at concentrations of 0.5 or 1 mM, no statistically significant differences in MDA content were observed compared with the control. However, treatment with 1 mM GABA resulted in a numerical reduction in MDA content by 8.91% and 7.52% in the first and second seasons, respectively. In contrast, the combined application of GABA and arsenic led to a marked decrease in MDA levels relative to arsenic treatment alone, indicating that GABA effectively mitigated arsenic-induced lipid peroxidation and oxidative membrane damage. Among the tested concentrations, 1 mM GABA was the most effective, reducing MDA content by 11.75% and 9.86% under 50 mg kg⁻¹ As in the first and second seasons, respectively.

**Fig. 5:**
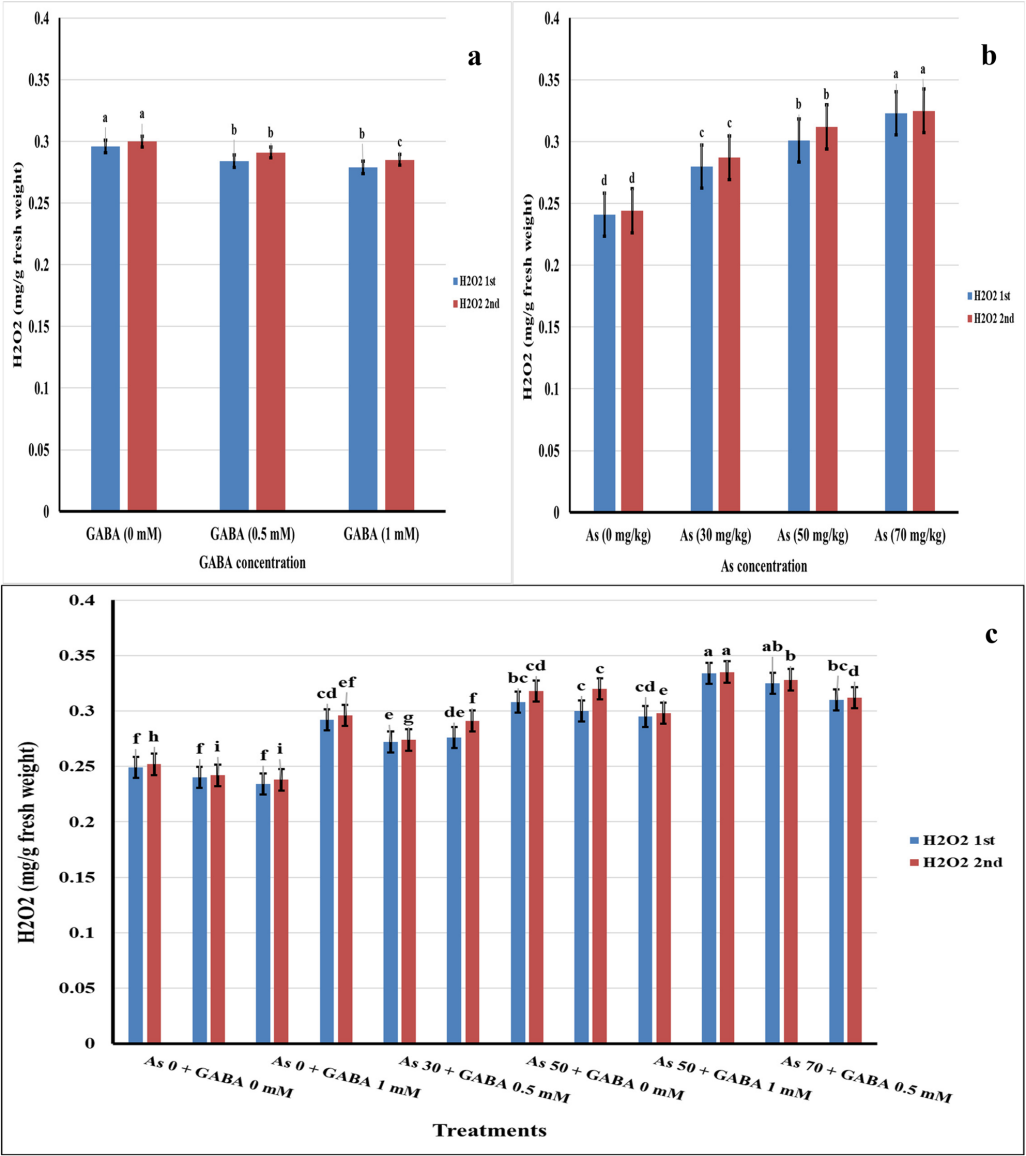
Effect of Arsenic Stress (**a**), GABA (**b**), and Their Interaction (**c**) on H_2_O_2_ content during the 2023 and 2024 seasons

Data presented in Fig. 5 show that hydrogen peroxide (H₂O₂) content increased significantly with increasing arsenic concentrations. At 70 mg kg⁻¹ soil arsenic, H₂O₂ levels rose by 25.4% and 24.9% in the first and second seasons, respectively, compared with the control, reflecting elevated oxidative stress.

Individual application of GABA resulted in a gradual reduction in H₂O₂ accumulation, with the greatest decrease observed at 1 mM GABA, where H₂O₂ levels declined by 5.7% and 5.0% in the first and second seasons, respectively. Under combined treatments, the application of 1 mM GABA with 70 mg kg⁻¹ As significantly lowered H₂O₂ content by 7.1% and 6.9% in the first and second seasons, respectively. This reduction highlights the enhanced capacity of GABA to strengthen antioxidant defense mechanisms. Overall, these findings suggest that exogenous GABA application, particularly at higher concentrations, effectively alleviates arsenic-induced oxidative damage by modulating redox homeostasis in plants under high arsenic stress.

### Superoxide dismutase isoenzyme

**Fig. 6:**
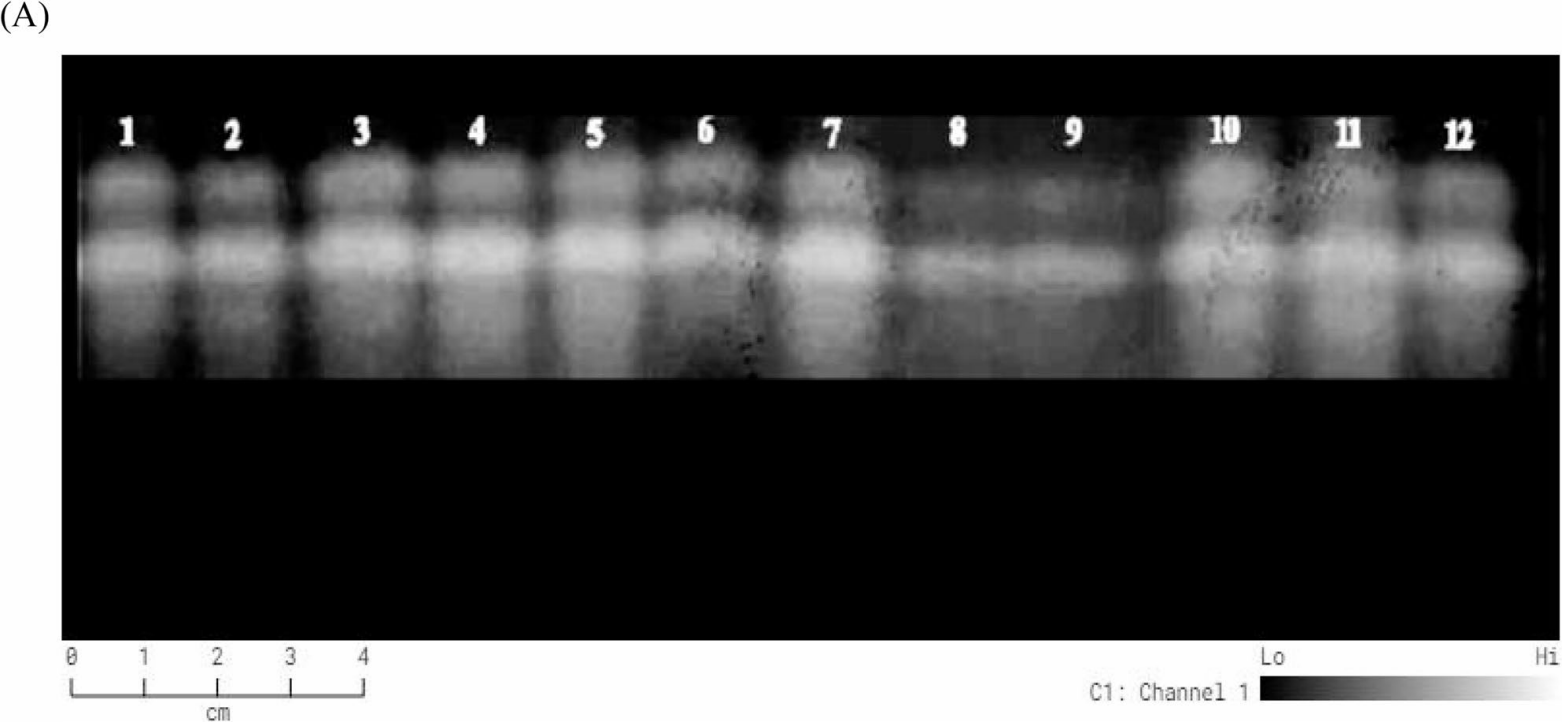
Isoenzyme banding patterns of Superoxide dismutase (**A**), effect of Arsenic (AS) and foliar application of gaba) and their interactions on Superoxide dismutase (SOD) isoenzyme of *Celosia argentea* var. *spicata *(1) As 0 mg/kg + 0 mM GABA, (2) As 0 mg/kg + 0.5 mM GABA, (3) As 0 mg/kg + 1 mM GABA, (4) As 30 mg/kg + 0 mM GABA, (5) As 30 mg/kg + 0.5 mM GABA, (6) As 30 mg/kg + 1 mM GABA, (7) As 50 mg/kg + 0 mM GABA, (8) As 50 mg/kg + 0.5 mM GABA, (9) As 50 mg/kg + 1 mM GABA, (10) As 70 mg/kg + 0 mM GABA, (11)As 70 mg/kg + 0.5 mM GABA, and (12)As 70 mg/kg + 1 mM GABA

Native-PAGE electrophoresis showed concise and consistent superoxide dismutase isoenzyme patterns in all investigated treatments in all assessed treatments (Fig. 6) and Table (). That it gave two bands at Rf (0.127 and 0.483) with a difference in intensity (Table S1), and no complete disappearance of isoenzyme bands was observed in any treatment, confirming that enzymatic activity was maintained across all experimental conditions.

**Fig. 7:**
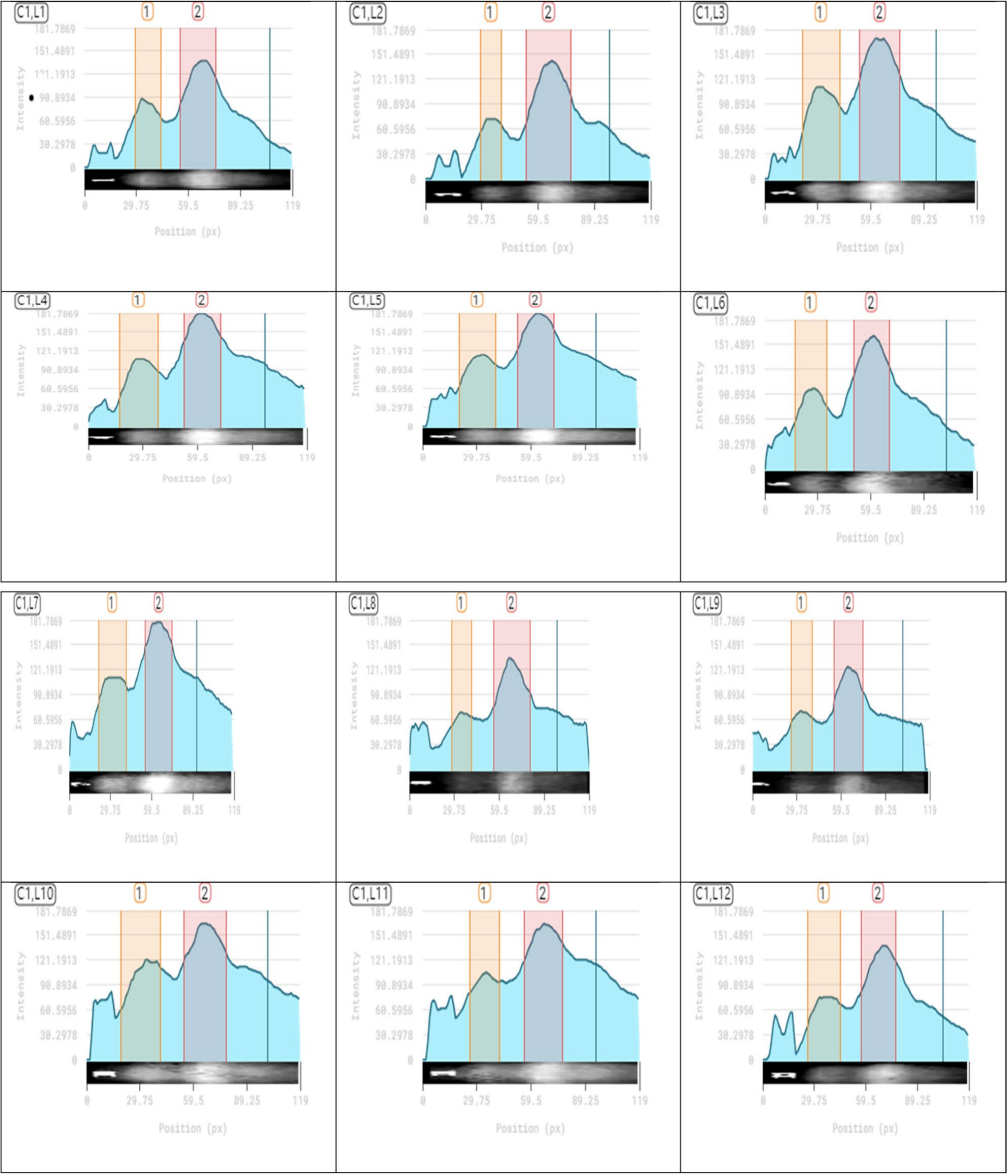
Densitometric Isoenzyme banding patterns of Superoxide dismutase (**A**), effect of Arsenic (AS) and foliar application of GABA) and their interactions on Superoxide dismutase (SOD) isoenzyme of *Celosia argentea* var. *spicata *(1) As 0 mg/kg + 0 mM GABA, (2) As 0 mg/kg + 0.5 mM GABA, (3) As 0 mg/kg + 1 mM GABA, (4) As 30 mg/kg + 0 mM GABA, (5) As 30 mg/kg + 0.5 mM GABA, (6) As 30 mg/kg + 1 mM GABA, (7) As 50 mg/kg + 0 mM GABA, (8) As 50 mg/kg + 0.5 mM GABA, (9) As 50 mg/kg + 1 mM GABA, (10) As 70 mg/kg + 0 mM GABA, (11)As 70 mg/kg + 0.5 mM GABA, and (12)As 70 mg/kg + 1 mM GABA

Our findings demonstrated that while SOD activity enhanced with increasing GABA levels when applied separately, arsenic stress alone caused a gradual increase in SOD expression and activity with rising arsenic concentrations. Certain SOD isoenzymes showed significant increases in integrated density under combined arsenic–GABA treatments, indicating increased sod activity; other treatments showed moderate reductions, indicating partial inhibition or redistribution of enzymatic activity among isoforms densitometry (Fig. 7). Overall, the isoenzyme patterns show that these treatments alter the *Celosia argentea var. spicata* antioxidant defense system. While the persistence of basal SOD activity throughout all treatments highlights its conserved and crucial role in stress tolerance, the selective enhancement of particular SOD isoenzymes probably enhances reactive oxygen species scavenging and supports cellular redox homeostasis.

## Discussion

Our study demonstrates the beneficial effect of γ-aminobutyric acid (GABA) against arsenic-induced toxicity in *Celosia spicata*, illustrating its diverse effects on vegetative and flowering traits, total sugar content, photosynthetic pigment, the total anthocyanin, the total proline, the total phenol, the total flavonoid, arsenic content in the shoots, roots and soil, MDA, H_2_O_2_, and SOD isoenzyme.

Arsenic is a naturally occurring toxic metalloid found everywhere in the environment and originates from both natural geological processes and human activities [22]. The two main forms of arsenic that plants can easily take up and cause toxicity are arsenate and arsenite [23].

The toxicity of arsenic stems from its chemical similarity to essential elements, such as phosphate and its high affinity for sulfur-containing groups in proteins. Arsenite is more damaging because it readily binds to these sulfur groups, inactivating critical enzymes involved in respiration and defence [24]. These negative impacts arise from increased production of reactive oxygen species (ROS), which induce oxidative stress and damage vital macromolecules. Arsenic also interferes directly with biomolecules such as thiol enzymes and ATP, hindering metabolic regulation and energy transfer. This disruption extends to the DNA repair system and cell cycle checkpoints, ultimately affecting plant cell division [25–27]. Previous studies on this subject indicate the harmful effects of arsenic on plant characteristics and functional efficacy. Arsenic negatively impacts plant productivity by competing with phosphate for transporter sites, hence diminishing phosphorus availability and carbon assimilation efficiency. It disrupts critical metabolic processes, compromises cellular membrane integrity, and impairs ion homeostasis.

### *Celosia spicata* performance under as stress

Our findings revealed that arsenic exposure has significant negative effects on the morphological and physiological characteristics of *Celosia spicata*. Arsenic contamination caused significant reductions in shoot and root traits, leaf area, and some flowering parameters, corroborating previous findings that As disrupts photosynthesis, mineral uptake, and energy metabolism [28, 29].

The observed decline in vegetative and floral traits with increasing As concentration reflects the inhibition of cell division, impairment of chloroplast ultrastructure, and decreased carbohydrate synthesis associated with oxidative stress. Our results agree with those of Talukdar [30] and Chandrakar et al. [31]. The availability of As in the soil limits the uptake of water and important minerals by root cells, thereby creating dehydration conditions within the plant, which is a major cause of leaf curling. Based on our data, we can said that the reduction of biomass in the presence of As was possibly an outcome of increased permeability of the cell membranes, consequently increasing the leakage of the cellular constituents/basic nutrients essentially required for energy generation and the optimum growth and development of plants [31]. Additionally, under arsenic stress, reduction/inhibition of growth may be related to reduce mitotic activity in the meristematic zone of the plant roots, which decreases the cell division rate in the apical meristem and reduces the expansion and elongation of newly formed cells. This agrees with [31, 32]. As the cellular turgor leads to an inhibition of cell enlargement in the elongation zone of the roots.

Interestingly, chlorophyll content increased under arsenic stress in our study. This finding contrasts with the usual reports of arsenic inhibiting chlorophyll production and photosynthesis [33, 34]. This unusual increase might reflect a stress-adaptive response under our experimental conditions. It may involve the upregulation of pathways for chlorophyll production or the accumulation of protective pigments to reduce mild oxidative stress. Alternatively, it could indicate that GABA plays a role in modulating metabolic processes, helping to stabilize photosynthetic pigments. This would allow plants to maintain or even improve photosynthetic capacity despite arsenic exposure. These results suggest that how plants respond to arsenic can depend on many factors, including species, dosage, and treatment interactions.

In some treatments, the chlorophyll levels were higher than expected under stress, which could reflect a GABA-induced modulation of metabolic flux that prioritizes the maintenance of photosynthetic pigments over secondary metabolite synthesis. These findings indicate that GABA not only mitigates arsenic-induced damage but may also optimize photosynthetic performance under metalloid stress.

The increase in photosynthetic pigments after treatment with gamma-aminobutyric acid indicates that it helped reduce chlorophyll degradation due to the presence of arsenic, thus enabling chloroplasts to function properly. It also preserved the content of chlorophyll a, b, and carotene, indicating improved photosynthetic efficiency and increased antioxidant protection within the chloroplasts. These findings are consistent with those of Shoaib et al. [8]., who reported that GABA improved pigment stability and redox balance in heavy-metal-stressed tomato plants.

Some previous studies [35–38] stated that some plants, when exposed to heavy elements their growth rate greater than that of plants that are not exposed, which is known as “hormesis”.we observed an increase in carotenoids at high arsenic concentrations, which suggests the activation of compensatory photoprotective mechanisms rather than an enhancement of photosynthetic performance. According to Foxx and Fort [39], and Liu et al. [40]., an increase in carotenoid content represents a crucial antioxidant and photoprotective response to arsenic-induced oxidative stress. Carotenoids play a crucial role in quenching singlet oxygen, scavenging free radicals, and facilitating non-photochemical quenching (NPQ), thereby preventing excessive excitation energy from harming PSII reaction centers.

One of the most notable impacts of arsenic exposure is the delay in flowering initiation. Arsenic stress often suppresses the synthesis and transport of key growth regulators, such as gibberellins and auxins, which play central roles in floral induction. Exposure to arsenic can change the expression of developmental genes and delayed flowering. For instance in rice, arsenic stress dramatically changes the expression of miR156, a regulatory RNA implicated in blooming and phase shift, suggesting developmental disturbance throughout flowering stages [31]. Arsenic also affects flower morphology and size. Stress-induced oxidative damage can compromise cell expansion and tissue differentiation within floral organs, leading to smaller inflorescences or reduced elongation of floral stalks [41]. In *Celosia argentea*, where ornamental value depends heavily on bright, well-developed inflorescences, such reductions are particularly detrimental. The accumulation of arsenic in reproductive tissues may further impair pigment formation, resulting in duller flower coloration due to disruptions in anthocyanin biosynthesis [42].

### Role of amino-GABA application in mitigating the harmful effects of as stress on *Celosia spicata* plants growth

GABA, a non-protein amino acid, helps plants counteract abiotic stresses by regulating the GABA shunt pathway, which replenishes succinate in the tricarboxylic acid (TCA) cycle during oxidative stress [43].

This metabolic flexibility probably helps keep ATP production going and stops too many ROS from forming, which makes the plant more resistant to stress, Also, the regulatory role of gamma-aminobutyric acid (GABA) in carbon and nitrogen metabolism and its ability to maintain osmotic and ionic balance in toxic environments [9, 44]. Our results proved that the application of gamma-aminobutyric acid (at 1 mM) assisted in the restoration of plant height, root development, spike formation, and total biomass at all As concentrations and the reduction of toxicity.

### GABA restricted the movement of Arsenic

Gamma-aminobutyric acid (GABA) restricted the movement of arsenic to the upper part of the *Celosia spicata*, therefore alleviating its harmful effects on plants according Michaeli and Fromm [45] they said that GABA application reduces the expression of Lsi-1 and Lsi-2 transporter genes, which are responsible for arsenite uptake and translocation in rice plants. ​ Araujo et al. [46]., is leads to decreased arsenic accumulation in roots and shoots, with long-term GABA exposure being more effective than short-term exposure.

### GABA enhance level of sugar, phenol and flavonoids content

GABA treatment reduced the levels of these compounds while increasing sugars and flavonoids. The results showed that GABA contributes to increased metabolic efficiency in plants, redirecting cellular resources from defense mechanisms to support growth and recovery processes. The increase in total sugar content provides further evidence of the positive role of GABA in carbon metabolism and osmotic regulation, a finding corroborated by Kumari et al. [9] in arsenic-exposed wheat. Furthermore, The results revealed that gamma-aminobutyric acid (GABA) acts as a signaling molecule and a key regulator of metabolic processes, enhancing a plant’s ability to withstand arsenic-induced stress through integrated regulation of the antioxidant defense system, improved nutrient uptake, and increased photosynthetic efficiency [47].

Furthermore, the marked improvement in the growth and flowering characteristics of GABA-treated *Celosia spicata* highlights its promising role as a sustainable and reliable biostimulant for improving the productivity of ornamental plants grown in heavy metal-contaminated environments.

### GABA role to reduce MDA, H_2_O_2_

The increased accumulation of malondialdehyde (MDA), hydrogen peroxide (H₂O₂), and superoxide dismutase (SOD) activity observed in our study is attributed to arsenic-induced inhibition of the tricarboxylic acid (TCA) cycle and mitochondrial respiration, which increases reactive oxygen species generation, resulting in oxidative stress in plants. This disruption induces hydrogen peroxide (H₂O₂) to accumulate, which leads to membrane lipid peroxidation, giving rise to elevated malondialdehyde (MDA) levels. In response, superoxide dismutase (SOD) activity increases as a major antioxidant defense against superoxide radicals. Excessive H₂O₂ buildup can overwhelm antioxidant enzymes, accelerating oxidative damage and hindering cellular metabolism and plant growth during arsenic stress [48].

GABA reduces oxidative stress cause of As by lowering H₂O₂ and MDA levels and improving antioxidant isoenzyme activities. This reduction decreases the need for high proline and phenolic accumulation in cells. Second, GABA serves as a central metabolic hub through the GABA shunt. It links carbon and nitrogen metabolism to energy production and cellular maintenance [49, 50]. When this pathway is activated, it may shift metabolic processes from secondary metabolite production to primary metabolism. This shift helps plants maintain growth and tolerate stress more effectively without relying on excessive osmolyte or phenolic accumulation [51]. Therefore, the reduced proline and phenolics in GABA-treated plants indicate both lower stress levels and a more strategic metabolism.

### The application of GABA significantly increased Superoxide dismutase isoenzyme activity

The application of GABA significantly increased the activity of the superoxide dismutase (SOD) isoenzyme in response to arsenic stress [52],, indicating its role in bolstering the antioxidative defence system. SOD prevents oxidative damage to cellular macromolecules by catalysing the dismutation of superoxide radicals (O₂•⁻) into hydrogen peroxide (H₂O₂) and molecular oxygen, thereby constituting the first line of defence against oxidative stress.‏.

## Conclusion

**Fig. 8:**
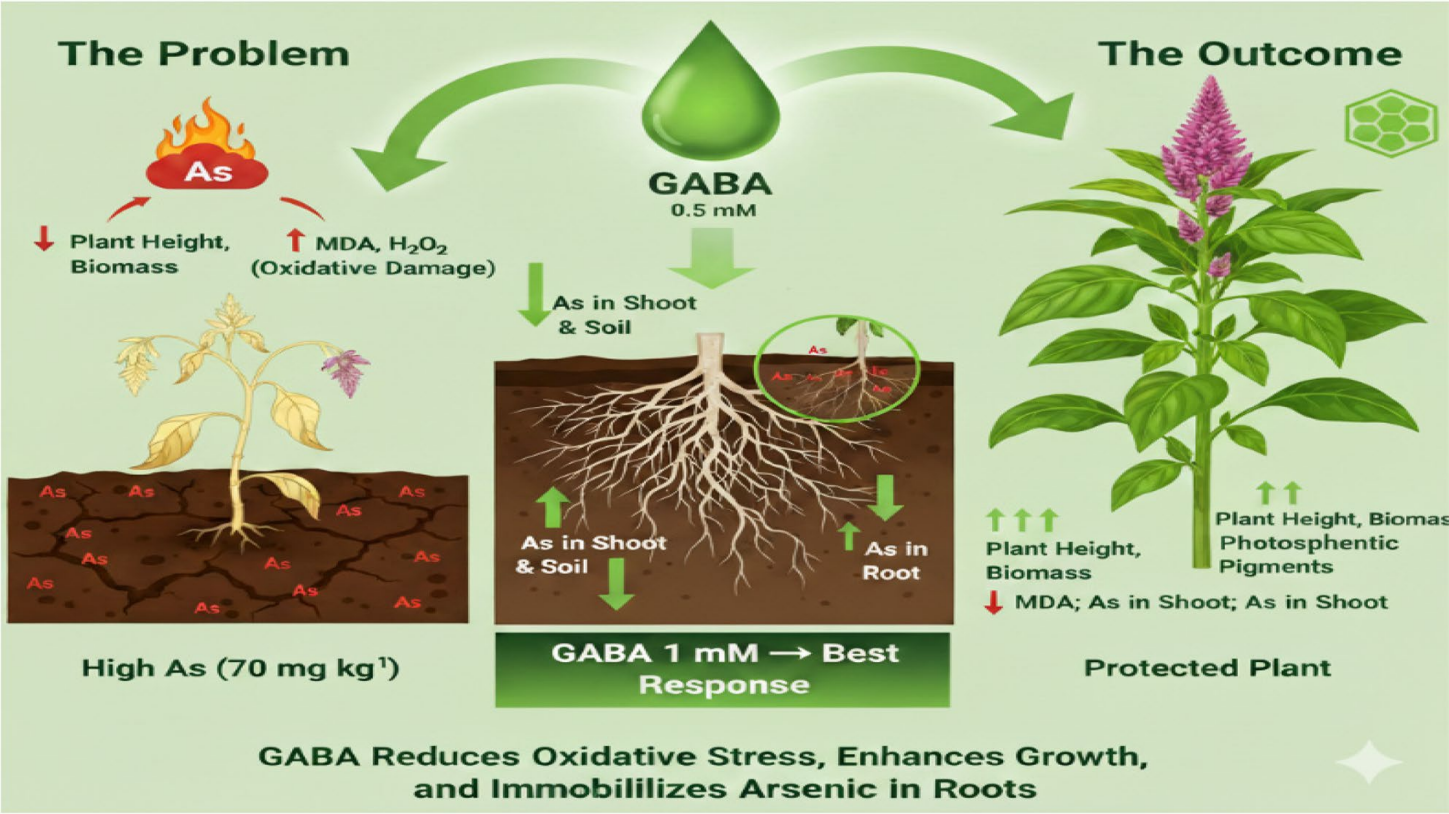
Conclusion of the mechanisms utilized by GABA to alleviate the impact of arsenic soil contamination on celosia plants

This study demonstrates that γ-aminobutyric acid (GABA), a natural, non-protein amino acid, is a highly versatile and environmentally friendly compound utilized in agriculture and food production, providing a sustainable alternative to chemical agents. It functions as a stress-reducing, bio-stimulating agent in plants, enhancing resistance to abiotic stress, and is produced safely through microbial fermentation. GABA had a positive impact role to mitigate the adverse effects of arsenic on vegetative and flowering traits by regulating a network of protective antioxidants (phenolic and flavonoids, MDA and H_2_O_2_), metabolic (SOD expression activity), osmotic adaptation (sugar contents, proline), and helps the plant immobilize the Arsenic in less sensitive tissues. GABA integrates multiple physiological responses that collectively enhance plant resilience under arsenic stress. These findings highlight GABA as an important regulatory molecule in plant stress tolerance and suggest its potential application as a strategic target for improving plant performance and sustainability in arsenic-contaminated agroecosystems. Our data indicate that GABA can play a meaningful role in restricting arsenic movement inside plants and reducing the oxidative damage associated with arsenic stress (Fig. 8). It is highly recommended to utilize GABA with flowering plants like *Celosia spicata* under metalloid stress to enhance their quality, along with environmentally safe disposal of such pollutants. Future work should use molecular techniques (gene expression of transporters, antioxidant enzymes, phytochelatin synthase) to validate the proposed mechanisms.

## Supplementary Information

Supplementary Material 1.

## Data Availability

The datasets generated and/or analyzed during the current study are included in this published.
