# Supplementary material for: Mitigating the harmful consequences of arsenic on celosia plants via the use of GABA
Source: BMC Plant Biol. 2026 Mar 13;26:637. doi: 10.1186/s12870-026-08384-2 (PMC13064408; doi:10.1186/s12870-026-08384-2)
Supplement: Supplementary file 1 — Supplementary Material 1. [file 12870_2026_8384_MOESM1_ESM.docx]

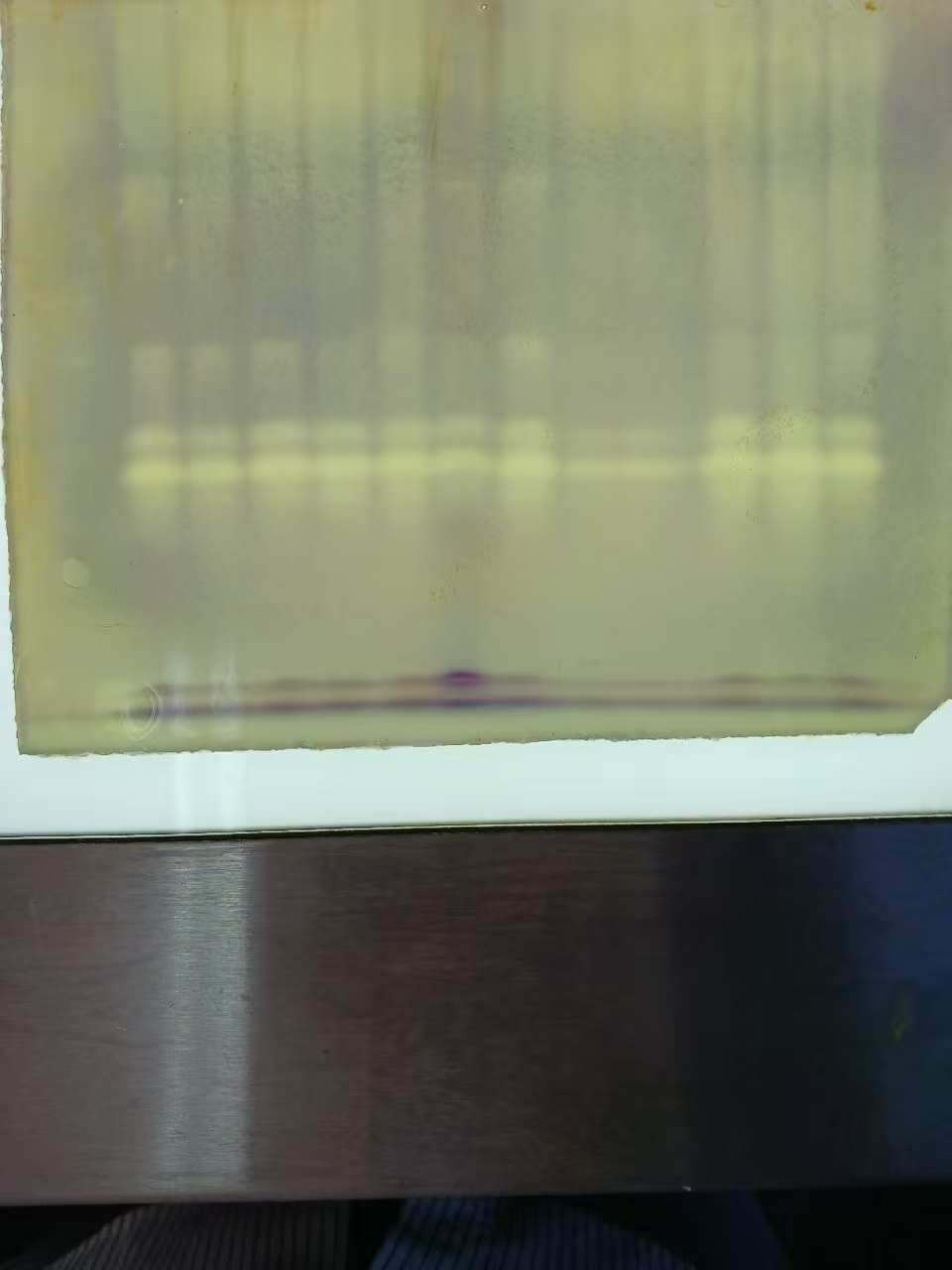

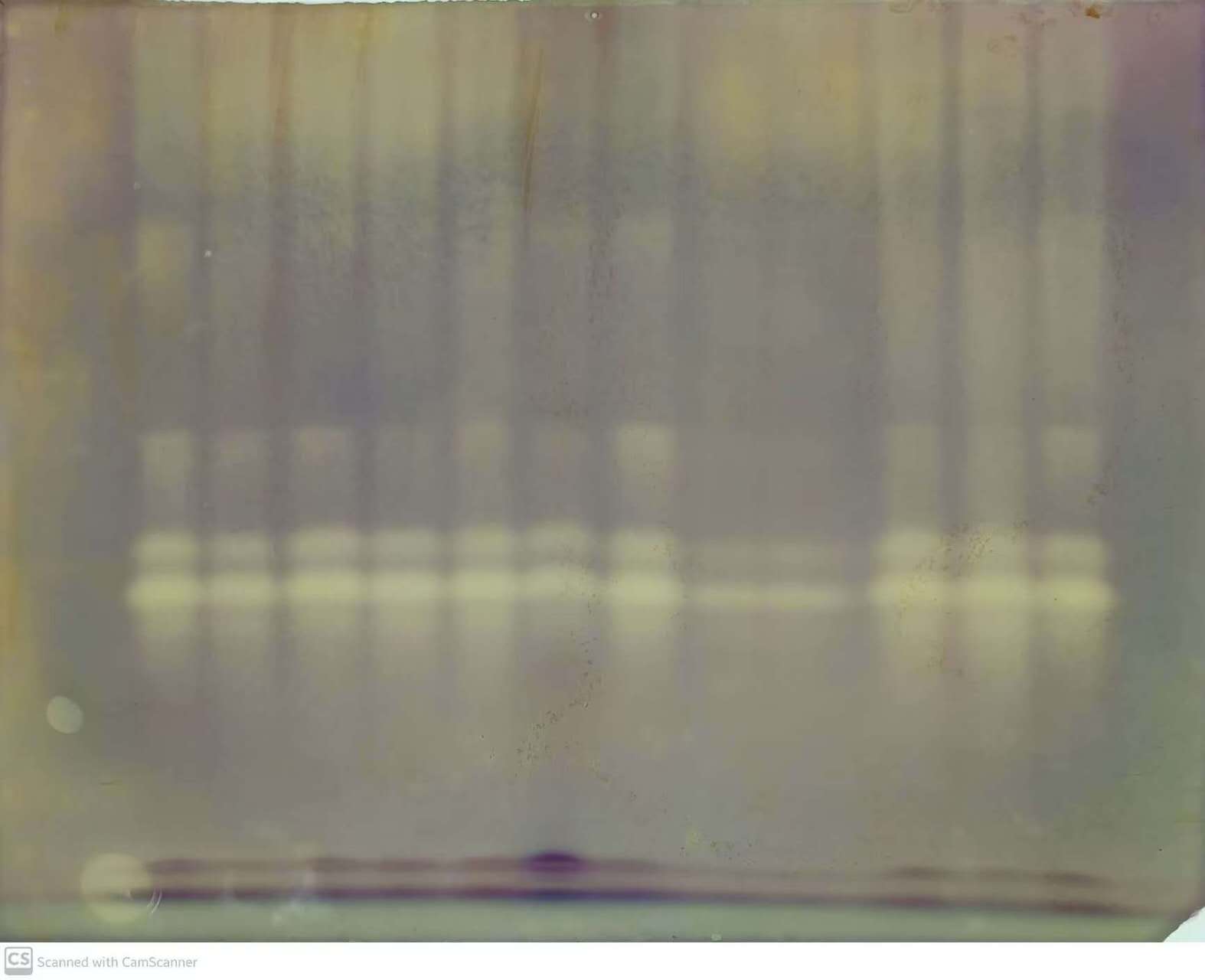
**Fig. 6.** Isoenzyme banding patterns of Superoxide dismutase (A), effect of Arsenic (AS) and foliar application of gaba) and their interactions on Superoxide dismutase (SOD) isoenzyme of Celosia argentea var. spicata (1) As 0 mg/kg + 0 mM GABA, (2) As 0 mg/kg + 0.5 mM GABA, (3) As 0 mg/kg + 1 mM GABA, (4) As 30 mg/kg + 0 mM GABA, (5) As 30 mg/kg + 0.5 mM GABA, (6) As 30 mg/kg + 1 mM GABA, (7) As 50 mg/kg + 0 mM GABA, (8) As 50 mg/kg + 0.5 mM GABA, (9) As 50 mg/kg + 1 mM GABA, (10) As 70 mg/kg + 0 mM GABA, (11) As 70 mg/kg + 0.5 mM GABA, and (12) As 70 mg/kg + 1 mM GABA.

**Table 2. Densitometric analysis of sod isoenzyme for celosia plants treated with As and/or GABA during**

|  | **lane signal** | **arsenic signal** | **arsenic ratio** | **normalize ratio** |
| --- | --- | --- | --- | --- |
| As 0 mg/kg + 0 mM GABA | 1941167 | 522578 | 0.269208162 | 1 |
| As 30 mg/kg + 0 mM GABA, | 1929493 | 524061 | 0.271605546 | 1.008905318 |
| As 50 mg/kg + 0 mM GABA | 2015390 | 543143 | 0.269497715 | 1.001075574 |
| As 70 mg/kg + 0 mM GABA | 2031446 | 547895 | 0.269706898 | 1.001852606 |
|  | **lane signal** | **gaba signal** | **salt-lane ratio** | **normalize ratio** |
| As 0 mg/kg + 0 mM GABA | 2817748 | 746082 | 0.264779533 | 1 |
| As 0 mg/kg + 0.5 mM GABA, | 2818714 | 667518 | 0.236816506 | 0.89439128 |
| As 0 mg/kg + 1 mM GABA | 2945817 | 701400 | 0.23810033 | 0.899239935 |
| As 30 mg/kg + 0 mM GABA | 2990299 | 697326 | 0.233196078 | 0.880717915 |
|  |  |  |  |  |
|  | **lane signal** | **treat signal** | **treat ratio** | **normalize ratio** |
| As 30 mg/kg + 0 mM GABA | 2031446 | 547895 | 0.269706898 | 1 |
| As 30 mg/kg + 0.5 mM GABA | 2043371 | 546882 | 0.267637154 | 0.992325951 |
| As 30 mg/kg + 1 mM GABA | 1986804 | 528511 | 0.266010638 | 0.98629527 |
|  | **lane signal** | **Treat signal** | **Treat ratio** | **normalize ratio** |
| As 50 mg/kg + 0 mM GABA | 2064672 | 552077 | 0.267392109 | 1 |
| As 50 mg/kg + 0.5 mM GABA | 1933362 | 510096 | 0.263838847 | 0.986711417 |
| As 50 mg/kg + 1 mM GABA | 1929527 | 510749 | 0.26470166 | 0.98993819 |
|  | **lane signal** | **salt signal** | **salt-lane ratio** | **normalize ratio** |
| As 70 mg/kg + 0 mM GABA | 2041065 | 537713 | 0.263447269 | 1 |
| As 70 mg/kg + 0.5 mM GABA | 1949054 | 540473 | 0.277300167 | 1.052583192 |
| As 70 mg/kg + 1 mM GABA | 1856667 | 521030 | 0.280626521 | 1.06520945 |
